# Understanding the Shift in the Microbiome of Composts That Are Optimized for a Better Fit-for-Purpose in Growing Media

**DOI:** 10.3389/fmicb.2021.643679

**Published:** 2021-04-07

**Authors:** Steffi Pot, Caroline De Tender, Sarah Ommeslag, Ilse Delcour, Johan Ceusters, Ellen Gorrens, Jane Debode, Bart Vandecasteele, Karen Vancampenhout

**Affiliations:** ^1^Division Forest, Nature and Landscape, Department of Earth and Environmental Sciences, KU Leuven, Geel, Belgium; ^2^Flanders Research Institute for Agriculture, Fisheries and Food (ILVO), Plant Sciences Unit, Merelbeke, Belgium; ^3^Department of Applied Mathematics, Computer Science and Statistics, Ghent University, Ghent, Belgium; ^4^PCS Ornamental Plant Research, Destelbergen, Belgium; ^5^Research Group for Sustainable Crop Production & Protection, Division of Crop Biotechnics, Department of Biosystems, KU Leuven, Geel, Belgium; ^6^Centre for Environmental Sciences, Environmental Biology, UHasselt, Diepenbeek, Belgium; ^7^Lab4Food, Department of Microbial and Molecular Systems, KU Leuven, Geel, Belgium

**Keywords:** compost, microorganisms, maturation, optimization, sustainable growing media, metabarcoding, phospholipid fatty acid, Biolog EcoPlates

## Abstract

Three characteristics are considered key for optimal use of composts in growing media: maturity, pH and organic matter content. Maturation is a critical step in the processing of composts contributing to compost quality. Blending of composts with chopped heath biomass, sieving out the larger fraction of composts and acidification of composts by adding elemental sulfur may be used either to increase organic matter content or to reduce pH for a better fit in growing media. While several studies have shown the effectiveness of these treatments to improve the use of composts in growing media, the effect of these treatments on the compost microbiome has merely been assessed before. In the present study, five immature composts were allowed to mature, and were subsequently acidified, blended or sieved. Bacterial and fungal communities of the composts were characterized and quantified using 16S rRNA and ITS2 gene metabarcoding and phospholipid fatty acid analysis. Metabolic biodiversity and activity were analyzed using Biolog EcoPlates. Compost batch was shown to be more important than maturation or optimization treatments to determine the compost microbiome. Compost maturation increased microbial diversity and favored beneficial microorganisms, which may be positive for the use of composts in growing media. Blending of composts increased microbial diversity, metabolic diversity, and metabolic activity, which may have a positive effect in growing media. Blending may be used to modify the microbiome to a certain degree in order to optimize microbiological characteristics. Acidification caused a decrease in bacterial diversity and microbial activity, which may be negative for the use in growing media, although the changes are limited. Sieving had limited effect on the microbiome of composts. Because of the limited effect on the microbiome, sieving of composts may be used flexible to improve (bio)chemical characteristics. This is the first study to assess the effects of maturation and optimization treatments to either increase organic matter content or lower pH in composts on the compost microbiome.

## Introduction

In horticulture, peat is still the most important resource for the production of growing media. The yearly extraction of peat for the horticultural sector in Europe amounts to 5.4 million tons (Parish et al., 2008). However, the sustainability of peat as a resource has been questioned for several reasons: valuable habitats for protected plant and animal species are lost when peat is extracted, and peat-based horticultural growing media represent a yearly emission of 4.5 million tons of CO_2_, long-distance transport not included (Parish et al., 2008). According to Vlaco (2009), each cubic meter of peat in growing media that can be replaced by a renewable source of biomass will result in a reduction of CO_2_ emission of 247 kg. Hence, the horticultural sector is in need of innovative growing media that reduce dependence on unrenewable resources.

To replace peat in growing media, the use of locally produced composts is promising, and steadily growing (Raviv, 2013). Some of these composts have certain physical and chemical properties similar to peat, making them suitable peat substitutes. Additionally, composts can have a high water-holding capacity, and provide nutrients for plants (Vandecasteele et al., 2018). Moreover, composts are characterized by high microbial activity that may stimulate plant growth and disease suppression (Hoitink et al., 1996; Noble and Coventry, 2005; Raviv, 2008). Diverse potential biocontrol agents present in composts, such as *Trichoderma*, *Pseudomonas*, *Pantoea* and *Bacillus* spp., are known to contribute to a biocontrol effect (Dukare et al., 2011; Chen et al., 2012; Antoniou et al., 2017; Lutz et al., 2020). This is in contrast to peat, which is considered to be poor in microorganisms, either beneficial or neutral, as compared to alternative growing media containing composts or other renewable materials, due to the recalcitrant chemical composition of *Sphagnum* biomass (Hoitink and Boehm, 1999). Successful substitution of peat in growing media therefore not only reduces emissions to the climate, but it also has the potential to reduce the use of chemical plant protection products.

The beneficial effects of composts are, however, highly dependent on the quality of the composts, and three characteristics are considered key for optimal use in growing media: maturity, pH and organic matter content (Vandecasteele et al., 2021). A first important factor contributing to compost quality is the degree of maturity (Bernal et al., 1998). Mature composts are characterized by a stable temperature, decrease in microbial activity and stable organic matter content (i.e., a low decomposition activity; Ryckeboer et al., 2003), and by the absence of phytotoxic compounds and pathogens (Hoitink et al., 1996). Second, pH is considered to be key for the optimal use of composts in growing media. Most composts are characterized by a neutral to alkaline pH, and are therefore unsuitable for direct use as growing media for many ornamental plants, with a desirable pH of 4.5 to 6 (Stoffella and Kahn, 2001; Raviv, 2005). Several methods are available to lower the pH of composts. A possible method is the application of acidifying chemicals such as elemental sulfur or sulfates before medium preparation (Martinez et al., 1988; Carrión et al., 2008; Irum et al., 2019). Under aerobic conditions, oxidation of elemental sulfur by sulfur-oxidizing bacteria, particularly *Thiobacillus* spp., yields sulfuric acid producing acidity (Madigan et al., 2012; Irum et al., 2019). Another method for compost acidification is to mix the composts with acidic materials when preparing growing media (Garcia-Gomez et al., 2002; Moore, 2005). An example of a good acidic material to blend in growing media is chopped heath biomass, which is a biomass residue generated from heathland restoration, produced by removing the above-ground biomass and part of the ecto-organic litter layer (Miserez et al., 2019). A third important factor contributing to an optimal use in growing media is organic matter content. Composts have lower organic matter content as compared to peat (Vandecasteele et al., 2021). Several methods are available to increase organic matter content in composts. Composts can be sieved, as larger fractions tend to be richer in relative organic matter content (López et al., 2002), or blended with materials with higher organic matter content, such as chopped heath biomass, which may also reduce the pH, as described above.

Several studies have investigated the effectiveness of the aforementioned treatments to improve composts for use in growing media, and their effect on plant growth (Garcia-Gomez et al., 2002; López et al., 2002; Mazuela et al., 2005; Carrión et al., 2008; Amberger-Ochsenbauer et al., 2017; Irum et al., 2019; Vandecasteele et al., 2021). However, studies that address the specific effects of these optimization treatments on the compost microbiome are more scarce, and it remains unclear what shifts these treatments induce in the composts microbiome. Several studies have shown that microbial communities are substantially altered during different phases of the composting process, including the maturation phase (Ghazifard et al., 2001; Insam and Bertoldi, 2007; Rebollido et al., 2008; Partanen et al., 2010; Mehta et al., 2014). Maturation has been shown to increase microbial diversity, mainly linked to decreasing compost temperatures (Ishii et al., 2000; Ryckeboer et al., 2003), but to decrease microbial activity and metabolic diversity, which may be linked to a decreasing availability of organic compounds (Beffa et al., 1996; Ryckeboer et al., 2003). Regarding community composition, Steel et al. (2018) reported an increase in biomass of actinobacteria and a decrease in biomass of Gram-negative bacteria during the maturation process, while Beffa et al. (1996) and Mehta et al. (2014) reported the diversity of mesophilic bacteria, including nitrogen-fixating, sulfur-oxidating, and nitrifying bacteria during maturation. Tang et al. (2006) showed an increase in the proportion of Actionobacteria during compost maturation. The bacterial genera *Bacillus*, *Flavobacterium*, *Pseudomonas*, and *Cellulomonas* have been shown to be dominant in the maturation phase of composts, while *Altamaria*, *Aspergillus*, *Bipolaris* and *Fusarium* have been shown to be the dominant fungal genera (Ryckeboer et al., 2003). Several studies have shown the presence of microorganisms that have the potential to suppress soil-borne diseases in mature composts, such as *Pseudomonas*, *Trichoderma*, and *Bacillus* (Dukare et al., 2011; Chen et al., 2012; Antoniou et al., 2017; Lutz et al., 2020). Carrión et al. (2008) studied the effect of acidification on the compost microbiome and reported a decrease in microbial activity and an increase in autotrophic bacteria upon the addition of elemental sulfur. To the best of our knowledge, no studies are available on the effects of blending and sieving of composts. In this study we aim to increase our understanding of the shifts in the microbiome induced by these specific optimization treatments and their relative importance using a combination of measures of community distribution, microbial diversity, biomass and functionality. If microbiological communities in composts stay relatively unaffected by these treatments, this will allow compost providers, growing media producers, and horticulturists to be flexible in adjusting (bio)chemical characteristics of composts using these treatments. On the other hand, these treatments may have a positive effect on the microbiology of composts, and they may therefore be used to directionally optimize microbiological characteristics in composts.

In the present study, we studied the effect of maturation and three optimization methods to either lower the pH or increase organic matter content of composts (blending, sieving and acidification) on the compost microbiome. The objective of this study is threefold. First, we aim to determine the main biological differences between the five mature composts and the effect of compost batch on the microbiome. Second, we aim to evaluate the effect of the preceding maturation on the microbiome of composts. Third, we aim to determine the effect of elemental sulfur addition, blending with chopped heath biomass, and sieving on the microbial communities of composts. The information generated will allow us to get insight in the role and the adjustability of the microbiome of composts, allowing a more targeted use in the production of sustainable growing media.

## Materials and Methods

### Dataset and Treatments

In this study, five batches of compost from different installations and feedstocks were used. Each of these five batches of compost is produced at another commercial compost production facility in Flanders, and is representative for the green and/or VFG (vegetable, fruit and garden) composts available in Flanders. All composts are produced from plant-based feedstocks (vegetable, fruit and/or garden waste): three green composts, one VFG compost, and one mixture of green and VFG compost.

Upon arrival at the test facility, these five batches of compost were not considered stable as they developed core temperatures exceeding 40°C and showed high oxygen uptake rate (OUR), CO_2_ flux and nitrogen immobilization. After an initial sampling of these immature composts, they were stored (i.e., matured) for an additional 30 days to increase stability. During this period, the composts were mixed regularly, and water was added when needed. After this maturation period, compost temperatures had decreased under 20°C, indicating compost stability. Stability indicators (OUR, CO_2_ flux and nitrogen immobilization) were significantly altered by the storage of the composts, and indicated that the effect of storage was the further stabilization of the composts. In this paper, we consider the composts that were stored in order to increase stability as matured composts. The five resulting matured composts were sampled.

**FIGURE 1:**
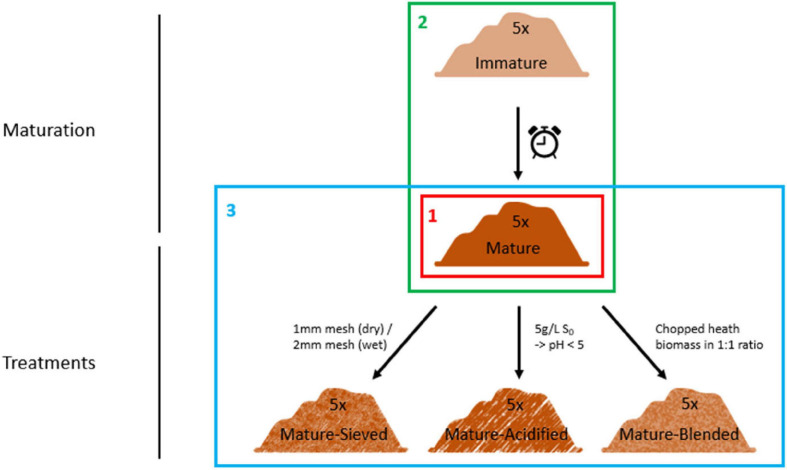
Set-up of the experiment. Five immature composts were matured by further storage. The matured composts were subsequently sieved or acidified by addition of elemental sulfur or blended with chopped heath biomass. These composts were characterized for their microbiological characteristics using three techniques: metabarcoding, PLFA analysis and Biolog EcoPlates. First, we determined the main microbiological differences between the 5 mature composts (Frame 1; see “Main Microbiological Differences Between the Five Mature Composts and the Effect of Compost Batch”) and the effect of compost batch. Second, the effect of compost maturation was evaluated, by comparing the microbiome of immature and mature composts in each compost batch (Frame 2; see “Effect of Compost Maturation”). Third, the effect of blending, sieving, and acidification on the microbiome was studied in each compost batch, for which the mature composts were used as a reference [Frame 3; see “Treatment Effects (Blending, Sieving and Acidification)”].

The resulting matured composts were then used for further treatment. They were well mixed and divided into three parts. The first part of the matured composts was blended in a 1:1 ratio with chopped heath biomass, which is an acidic material with a high C/P ratio and high organic matter content, in order to increase organic matter content and to decrease the pH. The second part of the matured composts was acidified by addition of elemental sulfur, targeting a pH of 5. Different concentrations of elemental sulfur were added depending on the initial pH of the composts. None of them was initially acidic. Each compost was mixed with 0.5 g/L elemental sulfur. If the targeted pH of 5 was not reached after two weeks of pH monitoring, additional sulfur was added in doses of 0.5 g/L. In order to increase the organic matter content, the finer fraction of the composts was sieved out in the third part of the matured composts. Four of the composts were sieved using a 2 mm sieve. One compost was sieved using a 1 mm sieve because it was more dry than the other four composts. For every treatment, one replicate per compost was sampled. This resulted in a total of 25 samples (5 immature, 5 mature, 5 blended, 5 sieved and 5 acidified composts). The experimental set-up is clarified in Figure 1.

For all 25 samples, three subsamples were taken: (1) 50 mL frozen at −20°C for metabarcoding analysis; (2) 150 mL frozen at −20°C, and later on freeze-dried before determination of total phospholipid fatty acids (PLFA); and (3) 150 mL stored at 4°C for Biolog.

Biochemical characteristics and suitability scores for the use in growing media of immature, mature and treated composts have been determined by Vandecasteele et al. (2021) (Supplementary Table 1).

### 16S and ITS2 Metabarcoding

#### Sampling and DNA Extraction

The 25 compost samples were each subsampled three times (250 mg per sample), resulting in three replicates of each compost. This resulted in 75 unique samples for DNA extraction. DNA was extracted from each sample using the DNeasy Powersoil Pro Kit (QIAGEN, Germantown, MD, United States), according to the manufacturer’s instructions. The DNA was stored at −20°C until use for metabarcoding, as described below.

#### 16S rRNA and ITS2 Gene Metabarcoding

Metabarcoding of the bacterial and fungal populations was done on the V3-V4 fragment of the 16S rRNA gene and the ITS2 gene fragment, respectively, as described in detail in De Tender et al. (2016a). Briefly, an amplification PCR was used to amplify fragments. The bacterial V3-V4 fragment was amplified using the primers S-D-Bact-0341-b-S-17 and S-D-Bact-0785-a-A-21, as described by Klindworth et al. (2013). For amplification of the fungal ITS2 fragment, an adapted forward primer of fITS7bis from Ihrmark et al. (2012) (GTGAATCATCRAATYTTTG) and the ITS4NGSr reverse primer (Tedersoo et al., 2014) were used. Fragments were extended with Illumina specific adaptors using a dual-index PCR. Mastermixes for all PCRs were prepared using the Kapa HiFi Hotstart ReadyMix (Kapa Biosystems, Wilmington, MA, United States) according to the manufacturer’s instructions. Each PCR step was followed by a PCR product clean-up using the CleanPCR reagent kit (MAGBIO, aitherburg, MD, United States). Final libraries were quality controlled by means of gel electrophoresis. Concentrations were measured using the Quantus double-stranded DNA assay (Promega, Madison, WI, United States). The final barcoded libraries were diluted to 10 nM and pooled. Resulting libraries were sequenced using Illumina MiSeq v3 technology (2x300 bp, paired-end) by Admera, United States, using 30% PhiX DNA as spike-in. Reads are available for download at the NCBI sequence read archive (SRA) under project numbers PRJNA624053, PRJNA692090 and PRJNA692114.

#### Sequence Reads Processing

Demultiplexing of the metabarcoding dataset and removal of the barcodes was performed by the sequencing provider. Primers were removed using Trimmomatic version 0.32 (Bolger et al., 2014). Adapters were already removed by the sequencing provider. For the ITS2-sequences, some adapters were still present and were removed using Cutadapt version 2.7 (Martin, 2011). Quality of the preprocessed sequences was checked using FastQC version 0.11.8 (Andrews, 2010). Further processing of the sequences was performed using the DADA2 pipeline version 1.12.1 (Callahan et al., 2015), as described in detail in Joos et al. (2020). Reads of a quality score less than or equal to two were truncated (truncQ = 2). For bacterial sequences, forward reads with more than three expected errors and reverse reads with more than five expected errors were filtered out (MaxEE = (3,5)). Forward reads were truncated after 255 bases and reverse reads were truncated after 240 bases. For fungal sequences, forward and reverse reads with more than two expected errors were filtered out (MaxEE = (2,2)), and 20 nucleotides were removed from the end of both forward and reverse reads. Next, error rates were estimated. The DADA2 algorithm uses a parametric model of errors introduced by PCR amplification and sequencing, which are estimated from the data itself. Thereafter, sequences were dereplicated and amplicon sequence variants (ASV) were inferred. Inferred ASVs were merged together. A sequence table was constructed with absolute counts of the resulting inferred ASVs. Chimeras were removed from the table. This procedure resulted in an average of 62251 ± 1420 reads per sample for the bacterial dataset and an average of 43830 ± 1344 reads per sample for the fungal dataset. Bacterial taxonomy was assigned using the SILVA database v132 (Quast et al., 2012; Yilmaz et al., 2013; Glöckner et al., 2017). Fungal taxonomy was assigned using the UNITE database v020219 (Nilsson et al., 2018).

#### Downstream Analysis

For further analysis, two sequence tables (bacterial and fungal) were constructed. For both bacterial and fungal ASV tables, ASVs with less than three counts per million in at least six samples were removed. Following analyses were done for both bacterial and fungal sequences.

First, the alpha diversity was studied. The Shannon diversity index was determined using the diversity function of the vegan package in R (version 2.5-6) (Oksanen et al., 2019). Following, the Shannon diversity index was analyzed in two steps. In the first step, the bacterial and fungal diversity was determined for the five mature composts to determine differences between the compost batches. In the second step, the effect of maturation, blending, sieving and acidification on diversity was determined in each compost batch using a Kruskal Wallis test with the mature composts as a reference. *P*-values lower than 0.05 were considered significant.

Second, beta diversity was studied. Absolute ASV counts of the 25 compost samples were transformed to relative abundances, and a dissimilarity matrix (based on the Bray-Curtis dissimilarity index) was calculated from the ASV table. Using the betadisper function, the homogeneity of the variances was checked on this dissimilarity matrix. The effect of treatment and compost batch was studied by doing a PERMANOVA analysis on the dissimilarity matrix. To visualize the observed differences, principal coordinate analysis (PCoA) on the dissimilarity matrix was done. Furthermore, PERMANOVA analysis and PCoA were used to determine treatment effects in each compost batch separately.

Third, relative abundances of phyla, families and genera in the 25 composts samples were determined. Absolute ASV counts were transformed to relative abundances and clustered at phylum, family and genus level. The effect of maturation and the three optimization treatments (blending, sieving and acidification) on abundance in each compost batch was tested using the edgeR package (version 3.28.0) (Robinson et al., 2010) as described in detail in De Tender et al. (2016b). The analyses were done upon clustering the bacterial and fungal ASV table with absolute sample counts at phylum, family and genus level. Normalization based on the trimmed mean of M-values (TMM) was applied to correct for differences in library size of the count table. A design matrix was defined based on the experimental design. The dispersion parameter was calculated. Following, a negative binomial model was fitted for every ASV and then combined. Likelihood-ratio tests were conducted on the contrast of the model parameters to assess differential abundances. *P*-values less than 0.05 and log2 fold changes smaller than −2 and larger than 0.5 were considered significant. Correction for multiple testing was included by adopting the Benjamini-Hochberg False Discovery Rate procedure.

### Biolog EcoPlates

Functional diversity of microbial communities in the 25 compost samples was determined using Biolog EcoPlates (Biolog, Inc., CA, United States), which have been used widely in soil sciences (Campbell et al., 1997; Preston-Mafham et al., 2002). Biolog EcoPlates allow determining a metabolic fingerprint of microbial communities by analyzing the characteristic reaction pattern of carbon metabolism at defined time intervals. They contain 31 of the most useful carbon sources for soil community analysis, including carboxylic acids, carbohydrates, amino acids, polymers, and amines/amides (Supplementary Table 2). A control well containing water is also included.

About 3 g of fresh material was added to 27,0 mL of Ringer Solution (Merck) and shaken for 30 minutes. Starting from that dilution (10^–1^), a ten-fold serial dilution was prepared until 10^–7^. Subsequently, each dilution was plated on Nutrient Agar (Merck) plates via spread plate method. Dilutions that resulted in 10 to 100 colonies were inoculated on Biolog EcoPlates. For each sample, three technical replicates were inoculated. Thereafter, the plates were incubated for 7 days at 25 °C. The optical density (OD590 nm) was measured directly after inoculation, after 1, 2, 3, 5 and 7 days with a microplate reader (VersaMax). To correct for background, readings of the initial well at time zero were subtracted from subsequent readings. Wells with a resulting optical density higher than 0.5 were considered as positive. Relative well optical density values that were negative or under 0.06 were set to zero (Classen et al., 2003).

The average well color development (AWCD) was calculated using formula 1. The AWCD is an indicator of total activity and can give an overall trend of metabolic activity of the microbial communities in time (Tanase et al., 2017).

(1) $$A\,W\,C\,D=\sum_{i=1}^{n}\frac{C_{i}-R}{n},$$

With C_i_ the optical density value of each reaction well at 590 nm, R the optical density value of the control well and n the number of wells.

For further analysis, data from day 7 were used because of the largest difference in optical density values. The optical densities of each well after 7 days were used to calculate the Shannon diversity index (H), substrate evenness (E) and Simpson diversity index (D):

(2) $$H=-\sum P_{i}.l\,n\,P_{i},$$

(3) $$P_{i}=\frac{C_{i}-R}{\sum \left(C_{i}-R\right)},$$

where P_i_ is the proportional optical density value of the ith well over total optical density of all wells of a plate,

(4) $$E=\frac{H}{l\,n\,S},$$

where S represents the total number of utilized carbon sources (i.e., substrate utilization richness),

(5) $$D=1-\sum P_{i}^{2}.$$

These indices reflect the metabolic functional diversity of the microbial communities of the composts.

Relative optical density values after 7 days were divided by the AWCD to minimize the influence of inoculum density differences between plates (Garland and Mills, 1991; Graham and Haynes, 2005).

### Phospholipid Fatty Acid (PLFA) Analysis

The 25 compost samples were used for phospholipid fatty acid (PLFA) analysis. Total PLFAs were isolated from 0.75 g freeze-dried material using phosphate buffer, chloroform, and methanol at a 0.9:1:2 ratio. Phospholipids separated by solid phase extraction were saponified to obtain free fatty acids, which were subsequently methylated using 0.2 M methanolic KOH to form fatty acid methyl esters (FAME). FAMEs were analyzed with a capillary gas chromatograph-flam ionization detector (Perkin Elmer Clarus 600, Perkin Elmer, Waltham, United States) with a SP-2560 column (100 m length × 0.25 mm ID, 0.20 μm film thickness, Supelco). The temperature program started at 75°C, followed by a heating rate of 10°C min^–1^ up to 180°C and a final heating rate of 2°C min^–1^ up to 240°C. PLFAs were identified by retention time using an external FAME (Restek 35077 Food Industry FAME Mix) and bacterial acid methyl ester (BAME) mix (Supelco 47080-U), and they were quantified by a C19:0 internal standard.

Seventeen PLFAs were selected because of their use of biomarker fatty acids for six distinct microbial groups: Gram-positive bacteria (i-C15:0, a-C15:0, i-C16:0, i-C17:0, 10Me-C16:0, 10Me-C18:0), Gram-negative bacteria (C16:1c9, C17:0cy, C19:0cy), bacteria (non-specific) (C14:0, C15:0, C16:0, C17:0, C18:0), actinomycetes (10Me-C16:0, 10Me-C18:0), fungi (C18:2n9,12) and mycorrhiza (C16:1c11), and they were summed up together with C18:1c9 to calculate total microbial biomass. To cope with the range of organic matter (OM) contents and bulk density of the different composts, PLFAs were expressed per g OM.

### Further Analysis of the Biolog EcoPlates and PLFA Analysis

Further analysis of the data from the Biolog EcoPlates and PLFA analysis was done in two steps. First, overall AWCD, functional diversity (calculated as Shannon diversity index) and absolute abundances of the PLFA biomarkers of the five mature composts were assessed to determine differences between the five compost batches. Second, the effect of maturation, blending, sieving and acidification on overall AWCD and functional diversity in each compost batch was determined using a Kruskal Wallis test with the mature composts as a reference. Since no replicates within each compost were available of the PLFA data, statistical analysis of these data was not possible.

To visualize differences in carbon source metabolization profiles and PLFA-based community composition, principal component analysis (PCA) was done on relative optical densities and on the biomass of microbial groups of the 25 compost samples. Loadings of the variables on the principal components were determined. A dissimilarity matrix was calculated from the relative optical densities and the biomass of microbial groups. Homogeneity of the variances was checked on this dissimilarity matrix using the betadisper function. PERMANOVA was used to determine significant shifts in the centroids and dispersion between compost batches and treatments. Furthermore, PERMANOVA analysis and PCA were used to determine treatment effects on the carbon source metabolization profiles in each compost batch.

All statistical tests were conducted in RStudio 1.2.5001 with R version 3.6.1.

## Results

The results are discussed in three sections (Figure 1). First, we determined if each compost batch consisted of a different microbial community (see “Main Microbiological Differences Between the Five Mature Composts and the Effect of Compost Batch”). Second, the effect of compost maturation was evaluated in each compost batch, by comparing the microbiome of immature and mature composts (see “Effect of Compost Maturation”). Third, the effect of blending, sieving, and acidification on the microbiome was studied in each compost batch, for which the mature composts were used as a reference [see “Treatment Effects (Blending, Sieving and Acidification)”].

### Main Microbiological Differences Between the Five Mature Composts and the Effect of Compost Batch

**FIGURE 2:**
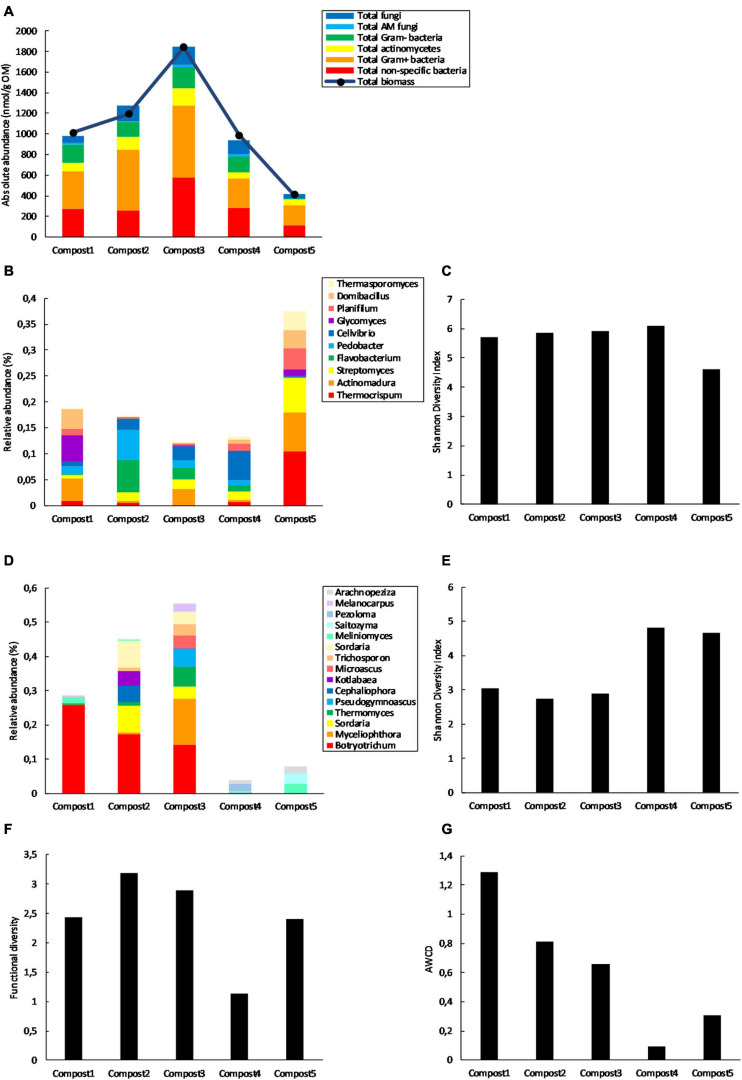
Main biological differences between the five mature composts (*n* = 1). **(A)** Absolute concentrations (nmol g^–1^ organic matter) of PLFA biomarkers specific for different microbial groups and total microbial biomass (nmol g^–1^ organic matter) in the five mature composts. **(B)** Relative abundance (percentages) of the 10 most abundant bacterial genera (16S V3-V4 region rRNA gene) in the five mature composts. **(C)** Bacterial diversity expressed as the Shannon Diversity Index in the five mature composts and calculated from the metabarcoding data. **(D)** Relative abundances (percentages) of the 15 most abundant fungal genera (ITS2 gene) in the five mature composts. **(E)** Fungal diversity expressed as the Shannon Diversity Index in the five mature composts and calculated from the metabarcoding data. **(F)** Functional diversity (calculated as the Shannon Diversity Index) of the five mature composts calculated from the Biolog data. **(G)** Microbial activity expressed as the average well color development (AWCD) of the five mature composts and calculated from the Biolog data.

The five composts used in this study have previously been characterized for their (bio)chemical and physicochemical properties in Vandecasteele et al. (2021) (Supplementary Table 1). In the present study, the five mature composts were screened for their microbiological characteristics, which are summarized in Figure 2. PLFA analysis showed considerable differences in microbial biomass between the composts, with the highest microbial biomass in compost 3 and the lowest in compost 5 (Figure 2A). Furthermore, 16S rRNA gene metabarcoding showed that the 10 most abundant bacterial genera represented between 13.2 and 22.9% of the bacterial community in compost 1, 2, 3, and 4, while they represented 41.2% of the bacterial community in compost 5 (Figure 2B). This is also reflected in the bacterial diversity, which is lower in compost 5 than in the other composts (Figure 2C). ITS2 gene metabarcoding showed that compost 1, 2, and 3 show similar composition in terms of most abundant fungal genera, while compost 4 and 5 show a different composition regarding the most abundant fungal genera (Figure 2D). Fungal diversity (calculated as the Shannon Diversity index from the Biolog Ecoplates data) is higher in composts 4 and 5 than in the other composts (Figure 2E). Furthermore, functional diversity is almost three times lower in compost 4 than in in compost 2, which has the highest functional diversity (Figure 2F). Metabolic activity is more than 13 times lower in compost 4 than in compost 1, which has the highest metabolic activity (Figure 2G).

To visualize differences in bacterial and fungal community distribution, principal coordinate analysis (PCoA) was used based on dissimilarity indices (Supplementary Figure 1). For bacteria, first and second principal coordinates (PCo) represented 20.1 and 16.8%, respectively, of the variance in the dataset. PCo1 represented variation between the different composts, while PCo2 represented variation between the different treatments [see “Effect of compost maturation” and “Treatment Effects (Blending, Sieving and Acidification)”]. PERMANOVA analysis showed that compost batch had a significant effect on the bacterial community composition (PERMANOVA; *P* = 0.00), which is also observed in Supplementary Figure 1. Similar, for the fungal community, compost batch had a significant effect (PERMANOVA; *p* < 0.001). The principal coordinates (PCo) represented 24.2 and 15.4%, respectively, of the variance in the dataset.

Principal component analysis (PCA) was used to differentiate microbial groups based on PLFA biomarkers (Supplementary Figure 2). The first and second principal components (PC) presented 62.5 and 22.3%, respectively, of total variability. The loadings of the biomass of microbial groups and total microbial biomass on the first two principal components are shown in Supplementary Figure 2. PC1 was mainly determined by the absolute abundance of fungi, Gram negative bacteria, non-specific bacteria (i.e., PLFAs not associated with specific bacteria), actinomycetes, and total microbial biomass. PC2 was mainly determined by the absolute abundance of arbuscular mycorrhizal fungi and Gram-positive bacteria. PERMANOVA showed no significant shifts in the composition of microbial communities based on PLFA biomarkers due to compost batch.

In order to evaluate and differentiate carbon source metabolization profiles, PCA was performed (Supplementary Figure 3). The first and second principal components (PC) presented 20.8 and 14.9%, respectively, of total variability. Loadings of the 31 carbon sources in the Biolog EcoPlates on the first two principal components are shown in Supplementary Figure 3. PC1 was mostly determined by pyruvic acid methyl ester, and it separated groups mainly based on their ability to utilize pyruvic acid methyl ester. A high score on PC2 was related to a high utilization of a group of carboxylic acids and amino acids. On the other hand, a lower score on PC2 was mainly related to the utilization of polymers, carbohydrates and amines. PERMANOVA showed no significant shifts in the carbon source metabolization profiles due to compost batch.

### Effect of Compost Maturation

#### Microbiome Composition and Shift

##### 16S V3-V4 region rRNA and ITS2 gene metabarcoding

**FIGURE 3:**
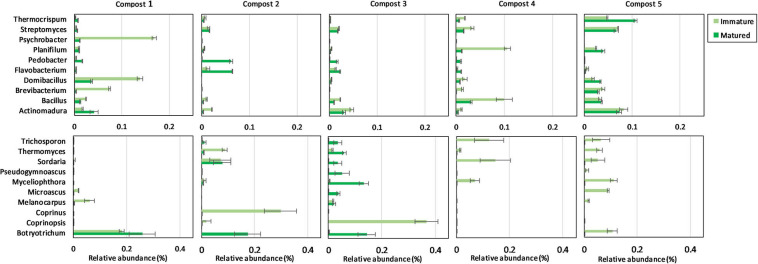
Microbial composition of immature and matured composts. On top: relative abundances (percentages) ± standard error of the ten most abundant bacterial genera (16S V3-V4 region rRNA gene) of immature and matured composts in the different compost batches. At the bottom: relative abundances (percentages) ± standard error of the ten most abundant fungal genera (ITS2 gene) of immature and matured composts in the different compost batches.

The ten most abundant bacterial and fungal phyla, families, and genera in immature and mature composts in each compost batch are shown in Supplementary Figures 4, 5 and Figure 3.

**FIGURE 4:**
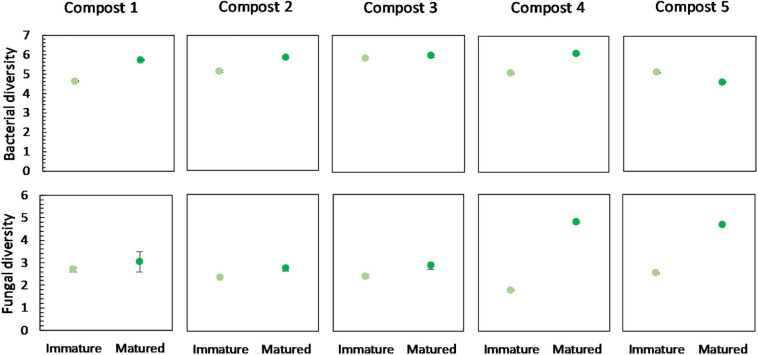
Microbial diversity in immature and matured composts in the five compost batches. On top: mean bacterial diversity ± standard error in the five compost batches, calculated as the Shannon diversity index from 16S V3-V4 rRNA gene sequencing data. At the bottom: mean fungal diversity ± standard error in the five compost batches, calculated as the Shannon diversity index from ITS2 gene sequencing data.

No significant differences were found in the bacterial diversity between immature and mature composts within each compost batch. However, in four of the five compost batches, a higher bacterial diversity is observed in matured composts as compared to immature composts (Figure 4). Similar, maturation resulted in higher fungal diversity in matured composts in each compost batch, although not significant (Figure 4).

**FIGURE 5:**
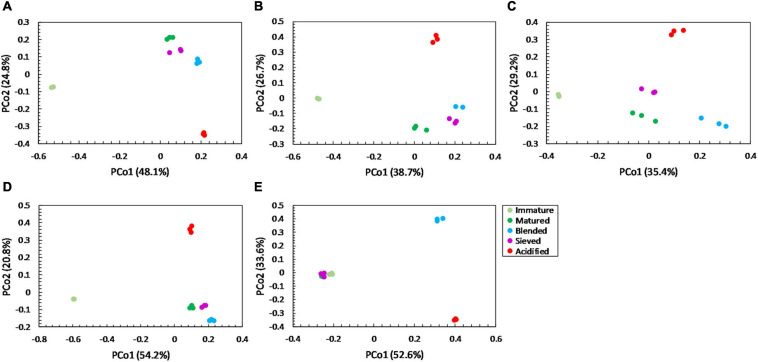
Major shifts in bacterial community distribution between immature, matured, blended, sieved and acidified composts in the five compost batches. – Principal Coordinate Analysis (PCoA) profile of pairwise community dissimilarity (Bray-Curtis) indices of bacterial (16S V3-V4 rRNA gene) sequencing data of the treated composts in **(A)** compost batch 1, **(B)** compost batch 2, **(C)** compost batch 3, **(D)** compost batch 4, **(E)** compost batch 5. Colors indicate the different treatments.

**FIGURE 6:**
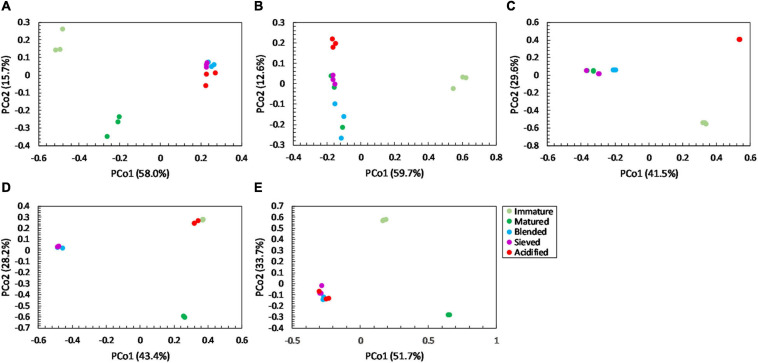
Major shifts in fungal community distribution between immature, matured, blended, sieved and acidified composts in the five compost batches. – Principal Coordinate Analysis (PCoA) profile of pairwise community dissimilarity (Bray-Curtis) indices of fungal (ITS2 gene) sequencing data of the treated composts in **(A)** compost batch 1, **(B)** compost batch 2, **(C)** compost batch 3, **(D)** compost batch 4, **(E)** compost batch 5. Colors indicate the different treatments.

For community distribution, PERMANOVA analysis showed that maturation had a significant effect on bacterial and fungal community distribution in each compost batch (PERMANOVA; *P* = 0.001 and *P* = 0.001, respectively, for each compost batch), which is also observed in the PCoA plots in Figures 5, 6.

When the bacterial and fungal communities were studied at taxonomic level, we saw that the relative abundances of several phyla (Supplementary Table 3), families (Supplementary Tables 4, 5) and genera (Tables 1A, 2A) were influenced by maturation. Phyla, families and genera that were significantly altered in at least four compost batches are discussed. For bacteria, the relative abundances of 10 phyla were significantly altered due to maturation, showing an increase for eight phyla and a decrease for two phyla. Furthermore, 25 bacterial families showed significant changes due to maturation: 24 increased and one decreased in relative abundance. The relative abundance of 24 bacterial genera significantly increased due to maturation in at least 4 compost batches. No fungal phyla were altered in relative abundance due to maturation. The relative abundances of two fungal families significantly increased due to maturation. Additionally, the relative abundances of 3 fungal genera significantly increased due to maturation in at least 4 compost batches.

**TABLE 1:** Major taxonomical changes in the bacterial communities.

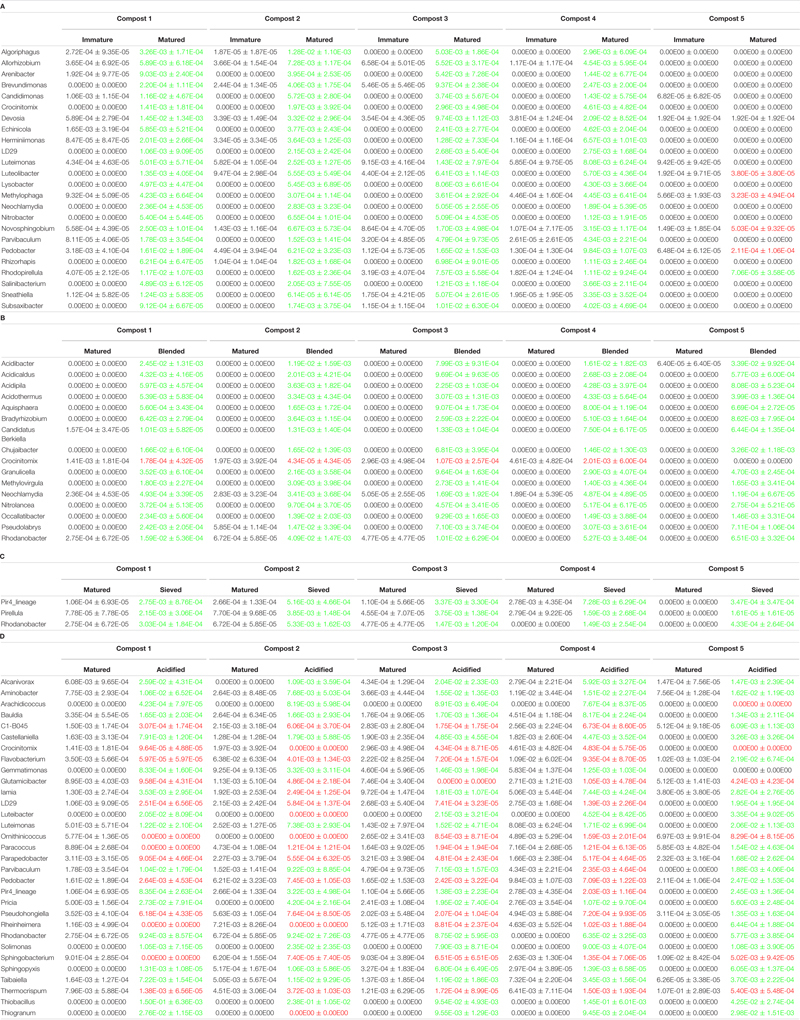

*(A) Relative abundances (percentages) ± standard error of bacterial genera (16S V3-V4 rRNA gene) that were significantly altered in mature composts as compared to immature composts in at least four compost batches. Green and red indicate a significant increase and decrease, respectively, in the relative abundance as compared to immature composts. (B) Relative abundances (percentages) ± standard error of bacterial genera (16S V3-V4 rRNA gene) that were significantly altered in blended composts as compared to matured composts in at least four compost batches. (C) Relative abundances (percentages) ± standard error of bacterial genera (16S V3-V4 rRNA gene) that were significantly altered in sieved composts as compared to matured in at least four compost batches. (D) Relative abundances (percentages) ± standard error of bacterial genera (16S V3-V4 rRNA gene) that were significantly altered in acidified composts as compared to matured in at least four compost batches. Green and red indicate a significant increase and decrease, respectively, in the relative abundance as compared to mature composts.*

**TABLE 2:** Major taxonomical changes in the fungal communities.

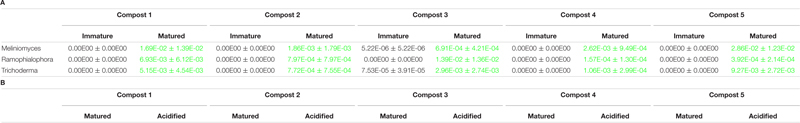

*(A) Relative abundances (percentages) ± standard error of fungal genera (ITS2 gene) that were significantly altered in mature composts as compared to immature composts in at least four compost batches. Green and red indicate a significant increase and decrease, respectively, in the relative abundances as compared to immature composts. (B) Relative abundances (percentages) ± standard error of fungal genera (ITS2 gene) that were significantly altered in acidified composts as compared to mature composts in at least four compost batches. Green and red indicate a significant increase and decrease, respectively, in the relative abundances as compared to matured composts.*

##### PLFA analysis

Because we only had one replicate per compost, we were unable to statistically test the effect of maturation on the absolute abundances of microbial groups and total microbial biomass. However, based on the PCA analysis in Supplementary Figure 2, it seems that there is no trend of an effect of maturation.

#### Microbial Activity

**FIGURE 7:**
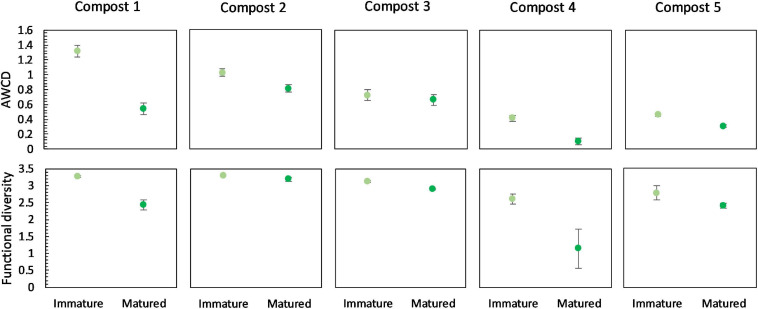
Metabolic characteristics of immature and matured composts in the five compost batches. On top: mean metabolic activity ± standard error in the five compost batches, expressed as mean AWCD (average well color development). Below: mean functional diversity ± standard error in the five compost batches, calculated as the Shannon diversity index from the Biolog Ecoplates data.

Overall, maturation had no significant effect on carbon source metabolization profiles in the different compost batches (Supplementary Figure 6). In none of the compost batches, a significant effect of maturation on functional diversity, calculated as the Shannon diversity index, was found. However, we can see a trend of a lower functional diversity in matured composts in each compost batch (Figure 7). Similar, in each compost batch, the mean metabolic activity, expressed as AWCD, was lower in matured composts than in immature composts, although not significant (Figure 7). According to the biochemical properties of carbon sources, the substrates in the Biolog EcoPlates were assigned into six categories, including carboxylic acids, carbohydrates, amino acids, polymers, miscellaneous and amines/amides (Tian-Yuan et al., 2014). The AWCD of the different carbon sources was determined and analyzed (Supplementary Figure 7). The results indicated no significant effect of maturation on the utilization of the different carbon sources in the different compost batches. Moreover, no trend was observed in the utilization of the different carbon sources.

### Treatment Effects (Blending, Sieving and Acidification)

#### Microbiome Shifts

##### 16S V3-V4 region rRNA and ITS2 gene metabarcoding

**FIGURE 8:**
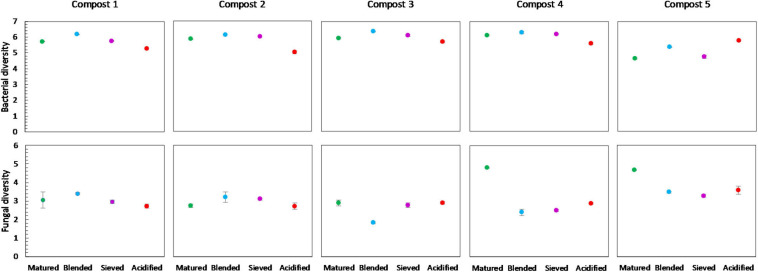
Microbial diversity in matured, blended, sieved and acidified composts in the five compost batches. On top: mean bacterial diversity ± standard error in the five compost batches, calculated as the Shannon diversity index from 16S V3-V4 rRNA gene sequencing data. At the bottom: mean fungal diversity ± standard error in the five compost batches, calculated as the Shannon diversity index from ITS2 gene sequencing data.

No significant differences in bacterial diversity due to treatments were observed in the different compost batches. However, some trends could be observed (Figure 8). In the five compost batches, blended composts showed a higher bacterial diversity than matured composts. Moreover, acidified composts showed a lower bacterial diversity in four of the five compost batches. In contrast, for the fungi no significant differences or trends in diversity due to treatments were observed in the different compost batches.

PERMANOVA analysis showed a significant effect of treatment on the bacterial community distribution in each compost batch (PERMANOVA; *P* = 0.001, for each compost batch). The PCoA in Figure 5 shows that acidification has the largest effect on bacterial community distribution in each compost batch, while sieving has the smallest effect. For fungi, a significant effect of treatment on the community distribution in each compost batch was shown (PERMANOVA; *P* = 0.001, for each compost batch). Acidification seems to have the largest effect on the fungal community distribution (Figure 6).

The relative abundances of several bacterial and fungal phyla (Supplementary Table 3), families (Supplementary Tables 4, 5), and genera (Tables 1B–D, 2B) were influenced by the optimization treatments. Phyla, families and genera that were significantly altered in at least 4 compost batches are discussed. For bacteria, the relative abundances of two phyla significantly decreased due to blending. Additionally, blending caused a significant change in the relative abundances of 25 bacterial families, with increases for 23 families and decreases in two families. The relative abundances of 16 bacterial genera were significantly altered in at least 4 of the compost batches: one genus decreased and 15 genera increased in relative abundance. Sieving caused a significant change in the relative abundances of two bacterial phyla: one increased and one decreased in relative abundance. The relative abundances of five families were significantly altered due to sieving: four increased and one decreased in relative abundance. The relative abundances of three bacterial genera significantly increased due to sieving in at least 4 compost batches. Furthermore, one bacterial phylum significantly increased in relative abundance due to acidification. The relative abundances of 26 families were significantly altered due to acidification: 17 increased and 9 decreased in relative abundance. The relative abundances of 31 bacterial genera were significantly altered due to acidification in at least 4 compost batches, with increases in 18 genera and decreases in 13 genera. For fungi, no phyla were significantly altered in relative abundance due to the treatments. Blending and sieving caused no significant changes in the relative abundance of fungal families or genera. Acidification significantly increased the relative abundances of two fungal families. The relative abundance of one fungal genus, *Thermomyces*, significantly increased due to acidification in at least 4 compost batches.

##### PLFA analysis

Because we only had one replicate per compost, we were unable to statistically test the effect of treatment on the absolute abundances of microbial groups and total microbial biomass. However, based on the PCA analysis in Supplementary Figure 2, it seemed that there is no trend of an effect of treatment.

#### Microbial Activity

**FIGURE 9:**
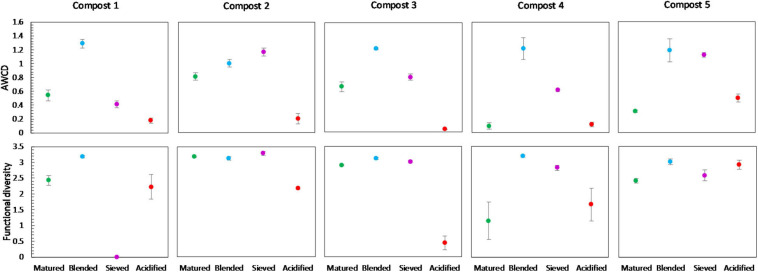
Metabolic characteristics of matured, blended, sieved and acidified composts in the five compost batches. On top: mean metabolic activity ± standard error in the five compost batches, expressed as mean AWCD (average well color development). Below: mean functional diversity ± standard error in the five compost batches, calculated as the Shannon diversity index from the Biolog Ecoplates data.

No significant shifts in the carbon source metabolization profiles communities due to blending, sieving or acidification were found by PERMANOVA analysis, as illustrated in the PCA plot in Supplementary Figure 6. Furthermore, no significant differences were found in the functional diversity or mean microbial activity, expressed as AWCD, due to treatment. However, some trends could be observed (Figure 9). Blended composts showed a higher AWCD than matured composts in the five compost batches, and a higher functional diversity than matured composts in four compost batches. Acidified composts showed a lower AWCD than matured composts in four compost batches. Sieved composts showed a higher AWCD than matured composts in four compost batches. Furthermore, the results indicated no significant effect of treatment on the utilization of the different carbon sources in the different compost batches. Moreover, no trend was observed in the utilization of the different carbon sources (Supplementary Figure 8).

## Discussion

In the present study, we assessed the effect of compost maturation and three compost optimization treatments - blending with chopped heath biomass, sieving and acidification by addition of elemental sulfur - on the microbiology of composts using 16S rRNA and ITS2 gene metabarcoding, PLFA analysis and Biolog EcoPlates. To our knowledge, this is the first time the effect of maturation and compost optimization treatments on the compost microbiome has been studied using the combination of these three techniques.

### Compost Batch Is Crucial for Microbiome

As expected, the five mature composts showed differences in their microbiological characteristics, such as microbial diversity, metabolic diversity, and metabolic activity. We noticed that the two composts, which previously received the highest suitability score to sustain plant growth (Vandecasteele et al., 2021), showed high microbial biomass, bacterial diversity, and functional diversity as compared to other composts. The composts with the lowest suitability score showed the lowest microbial biomass, bacterial diversity, and microbial activity. Additionally, bacterial and fungal community distribution significantly differed between the five composts. Microbial communities of the composts were analyzed at genus level, which revealed the absence of genera known to include human pathogens (such as *Salmonella*, *Escherichia*, *Klebsiella*, *Shigella*, and *Enterobacter*) or plant pathogens that can infect the plant roots via the growing medium (such as *Verticillium*, *Rhizoctonia*, *Fusarium*, *Pythium*, *Phytophthora*, *Sclerotinia*, and *Plasmodiaphora*). Since the use of composts in horticulture may pose a risk for the environment by the potential presence of human and plant pathogens (Jones and Martin, 2003), the absence of both human and plant pathogens is an important requirement for the further use of composts in growing media. The composts used in this study meet this requirement and therefore do not pose a risk for disease transmission when used in growing media in the horticultural sector. Overall, these composts show diverse microbiological characteristics.

Moreover, compost batch was shown to be the most important to determine bacterial and fungal community composition. Lutz et al. (2020) also state that microbial community composition is highly dependent on the initial characteristics of the composts, including the compost material. A previous study by Vandecasteele et al. (2021) showed that optimizing chemical properties of composts for a better fit in growing media was only successful if composts initially had a good suitability score.

### Maturation Increases Microbial Diversity and Favors Beneficial Microorganisms

Maturation had a significant effect on bacterial and fungal community distribution. Within each compost batch, maturation had the strongest effect on the bacterial community distribution. Analysis of the microbial communities at genus level showed a significant increase in genera known to include plant growth promoting rhizobacteria (PGPR) and fungi (PGPF) due to maturation, including bacterial species belonging to the genera *Nitrobacter* and *Pedobacter*, and fungal species belonging to the genus *Trichoderma*. Several studies have shown beneficial effects of these PGPR and PGPF on plant growth and health (Hyakumachi, 1994; de Boer et al., 2007; Anderson and Habiger, 2012; Berendsen et al., 2012; Ozimek et al., 2018; Johnson et al., 2019). This is in accordance to other studies that have shown the presence of microorganisms that may have the potential to support disease suppressiveness in composts (e.g., Dukare et al., 2011; Chen et al., 2012; Antoniou et al., 2017). Furthermore, a trend of increased bacterial and fungal diversity in matured composts was observed, which confirms previous studies (Ishii et al., 2000; Ryckeboer et al., 2003). The higher microbial diversity in matured composts may be considered to be positive for the use in growing media, as this may outcompete pathogens from growing media by niche saturation, leading to a higher disease suppressiveness (Chaparro et al., 2012; Fliessbach et al., 2009; van Elsas et al., 2012; Bongiorno et al., 2019).

In terms of microbial biomass, Steel et al. (2018) reported an increase in biomass of actinobacteria and a decrease in biomass of Gram-negative bacteria during maturation. However, in the present study microbial biomass seemed not affected by maturation.

Carbon source metabolization profiles were not affected by maturation either. However, maturation did decrease metabolic diversity in the five compost batches. A high metabolic diversity in immature composts may indicate an efficient decomposition of organic matter. The lower metabolic diversity in matured composts may indicate a higher stability, as the decomposition activity neared completion. Although not significant, the present study showed a decrease in microbial activity, measured as AWCD, due to maturation. High microbial activity in immature composts can also be related to instability of the composts (Ryckeboer et al., 2003; Ceustermans et al., 2010).

Maturation was shown to have an important effect on the compost microbiome, which is in accordance with Insam and Bertoldi (2007). Overall, the effect of maturation on the microbiome of composts seems to be positive, which is in correspondence to the positive effects of maturation on biochemical characteristics and compost stability (Vandecasteele et al., 2021).

### Blending and Acidification Have a Higher Impact on the Microbiome Than Sieving

Blending only had a limited effect on bacterial and fungal community distribution, with only a limited number of abundant taxa altered in relative abundance. However, in the five compost batches, a trend of increased bacterial diversity in blended composts was observed, which may be the result of the introduction of bacteria specific for the chopped heath biomass. Previous research showed specific microbiological characteristics of chopped heath biomass, such as higher fungal biomass, lower biomass of actinomycetes, lower biomass of Gram-positive bacteria and higher fungi:bacteria ratio than in composts (Vandecasteele et al., 2021). As mentioned before, a higher microbial diversity may have a positive effect on disease suppression in growing media (Fliessbach et al., 2009; Chaparro et al., 2012; van Elsas et al., 2012; Bongiorno et al., 2019). Furthermore, a trend of increased metabolic diversity and activity in blended composts was observed, which may again be linked to the introduction of microorganisms specific to the blended chopped heath biomass (Vandecasteele et al., 2021). An active and metabolic diverse microbial community may be less susceptible for invasion by other microorganisms and may be more suppressive to pathogens (Chen et al., 1988; Fliessbach et al., 2009). On the other hand, high microbial activity can also be related to instability of the composts as stability is based on measuring microbial decomposition activity in composts (Ryckeboer et al., 2003; Ceustermans et al., 2010). Biochemical indicators for stability did not show an increase in compost instability due to blending (Vandecasteele et al., 2021). Therefore, we can assume that the increase in metabolic activity due to blending, seen in this study, is not related to compost instability.

Acidification seemed to have the largest effect of the three optimization treatments on bacterial and fungal community distribution. Several taxa were altered in their relative abundance due to acidification. Analysis of the microbial communities at genus level showed an increase in sulfur-oxidizing bacteria due to acidification, such as bacteria belonging to the genera *Thiobacillus*, *Thiogranum* and *Halothiobacillus*. This is in accordance with the study by Carrión et al. (2008) that showed an increase in autotrophic bacteria. Sulfur-oxidizing bacteria are responsible for the oxidation of the added elemental sulfur to sulfuric acid, which produces acidity and lowers compost pH (Madigan et al., 2012; Irum et al., 2019). Moreover, acidification decreased bacterial diversity in the five compost batches. Acidification has also been shown to decrease bacterial diversity in soil (Zhang et al., 2015). As mentioned before, previous research has shown that a lower microbial diversity may increase potential invasion by pathogens (Fliessbach et al., 2009; Chaparro et al., 2012; van Elsas et al., 2012; Bongiorno et al., 2019). Furthermore, acidified composts showed a lower AWCD in the majority of the compost batches. As mentioned before, microbial community with low microbial activity may be more susceptible for invasion by other microorganisms, such as pathogens (Chen et al., 1988; Fliessbach et al., 2009). Low microbial activity may also be related to higher stability of the composts (Ryckeboer et al., 2003; Ceustermans et al., 2010). Vandecasteele et al. (2021) showed that acidification resulted in higher stability of composts. The decreased microbial activity in acidified composts may therefore be related to increased stability of the composts.

In contrast to the other treatments, which are mainly microbial controlled treatments, sieving is merely a physical intervention, resulting in a fraction with higher organic matter content than the initial material. As expected, we see no to limited effects on the microbial communities due to sieving as compared to the other treatments. Sieving only had a limited effect on community distribution, with only a limited number of taxa significantly altered in relative abundance. Microbial diversity or biomass were not affected by sieving. Sieving did cause a small increase in microbial activity in the majority of the compost batches. Previous research did not show an effect of sieving on compost stability (Vandecasteele et al., 2021). Therefore, this increase in microbial activity is probably not related to a decrease in stability.

Microbiological communities in composts stay relatively unaffected by sieving, which allows compost providers, growing media producers and horticulturists to be flexible in using this treatment to improve (bio)chemical characteristics of composts. Acidification seems to have a large effect on the composts microbiome, and may potentially be negative for the use in growing media, although the changes are limited. Blending seems to have a large effect on the composts microbiome, and it seems to be positive for the use in growing media. Blending may therefore be used to modify the microbiome of composts to a certain degree in order to optimize microbiological characteristics. However, at this moment, no targets for microbiological characteristics for growing media are defined, making it difficult to determine if observed changes are more or less optimal. Therefore, container experiments where treated composts are used in growing media are necessary to confirm our hypothesis on the effect of these treatments on plant health and disease suppressiveness.

## Conclusion

In the present study, we demonstrated that compost batch is more important than maturation or further optimization treatments to determine bacterial and fungal communities. However, compost maturation increased microbial diversity, and favored beneficial microorganisms. The effect of maturation on the microbiome of composts may be positive for the use in growing media, which corresponds to the positive effect of maturation on biochemical characteristics and compost stability. The effect of blending seems to be positive for the use in growing media, with increasing microbial diversity and metabolic diversity and activity. Therefore, blending may be used to modify the microbiome to a certain degree in order to optimize microbiological characteristics. Acidification may be negative for the use of composts in growing media, with decreased bacterial diversity, and microbial activity, although the effect seems limited. Sieving seemed to have no to limited effect on the microbiome of composts. Because of the limited effect on the microbiome, sieving of composts may be used flexible to improve (bio)chemical characteristics of the composts. This is the first study to assess the effects of maturation and optimization treatments to either increase organic matter content or lower pH in composts on the compost microbiome. Container experiments may confirm the effect of the use of treated composts in growing media on plants.

## Data Availability Statement

The datasets presented in this study can be found in online repositories. The names of the repository/repositories and accession number(s) can be found below: https://www.ncbi.nlm.nih.gov/, PRJNA624053; https://www.ncbi.nlm.nih.gov/, PRJNA692090; https://www.ncbi.nlm.nih.gov/, PRJNA692114.

## Author Contributions

JD, BV, ID, and KV were involved in the design of the study. CD, JD, BV, KV, and JC supervised the study. SO conducted the metabarcoding and PLFA analysis. EG conducted the Biolog EcoPlates experiment. SO and SP conducted the bio-informatics of the NGS data. SP conducted the statistical analysis of the data and wrote the first draft and finalized the manuscript. All authors contributed to the writing of the manuscript and approved submission.

## Conflict of Interest

The authors declare that the research was conducted in the absence of any commercial or financial relationships that could be construed as a potential conflict of interest.
