# Supplementary material for: Understanding the Shift in the Microbiome of Composts That Are Optimized for a Better Fit-for-Purpose in Growing Media
Source: Front Microbiol. 2021 Apr 7;12:643679. doi: 10.3389/fmicb.2021.643679 (PMC8059793; doi:10.3389/fmicb.2021.643679)
Supplement: Supplementary file 1 [file Data_Sheet_1.PDF]

Supplementary Tabel S1. Biochemical characteristics of the 25 composts. (VFG compost: vegetable, fruit and garden compost, BD: biodegradability, Nimmob: N immobilization, OUR: oxygen uptake rate, Cwater: water-extractable C, Nmin: mineral N = NO_3–N + NH_4–N, OM: organic matter, suitability score: score for chemical properties and stability for suitability in growing media).

**Compost batch**	**Description**	**Treatment**	**pH-H_2O**	**BD**	**Cumulative CO_2 release (mol CO_2/kg OM)**	**NO_3-N (mg/l compost)**	**NH_4-N (mg/l compost)**	**Nim mob (%)**	**OUR (mmol O_2/kg OM/hr)**	**Hemi-cellulose (%/OM)**	**Cellulos e (%/OM)**	**Lignin (%/OM)**	**Cwater (mg/l compost)**	**N-min (mg/l compost)**	**Suitability score**
1	VFG compost	Immature	9.15	1.11	2.79	5.00	401	50.2	8.10	9.42	23.3	29.5	559	400	4
		Matured	8.45	0.95	1.08	242	122	-58.3	2.40	9.73	23.7	35.4	309	364	6
		Blended	7.42	1.11	0.68	157	85.6	-4.40	1.70	12.1	24.4	33.0	256	242	9
		Acidified	5.5	0.94	0.45	332	7.60	5.00	2.40	9.38	17.9	29.1	159	340	8
		Sieved	8.25	1.18	0.09	280	5.00	1.36	2.40	12.5	26.2	32.9	322	280	6
2	green compost	Immature	8.85	1.00	2.49	5.00	61.6	7.06	3.80	11.7	26.5	38.4	303	61.6	9
		Matured	8.06	0.98	1.15	77.2	7.70	-38.0	2.20	9.80	24.2	34.7	265	84.9	9
		Blended	6.55	0.95	0.66	95.0	57.0	6.30	2.00	10.2	19.3	31.0	253	152	13
		Acidified	5.18	0.93	0.52	5.00	33.2	2.00	2.10	11.8	23.7	38.1	115	33.2	10
		Sieved	8.07	1.21	1.20	79.7	5.00	42.0	3.00	12.2	30.3	35.1	235	79.7	8
3	mixture of VFG and green compost	Immature	8.79	1.21	2.39	5.00	180	27.3	5.80	14.8	22.8	31.1	441	216	8
		Matured	8.26	1.00	1.17	171	76.5	-34.0	5.40	13.0	20.3	33.2	242	247	9
		Blended	7.39	1.08	0.72	26.6	92.6	10.0	2.60	14.1	23.1	34.4	324	119	12
		Acidified	4.99	1.04	0.56	220	31.8	9.50	2.50	15.8	23.1	37.3	132	252	12
		Sieved	8.1	1.05	0.78	201	5.00	-6.00	0.20	12.8	22.1	33.4	245	201	9
4	green compost fraction 0-15 mm	Immature	8.01	0.95	3.63	5.00	179	50.4	11.7	9.70	20.2	31.4	679	179	3
		Matured	8.26	1.04	1.33	225	6.10	-27.9	3.00	15.6	22.3	36.5	273	231	8
		Blended	7.39	0.99	0.58	101	54.1	0.00	1.80	11.6	20.9	32.9	240	155	12
		Acidified	4.61	0.91	0.39	63.6	10.3	3.40	1.20	11.4	20.6	35.3	130	73.9	8
		Sieved	9.09	1.17	1.06	85.8	5.00	13.8	3.70	12.8	26.0	33.1	275	85.8	9
5	green compost	Immature	8.88	1.16	1.68	5.00	181	51.9	7.20	10.8	24.7	30.5	505	181	4
		Matured	8.88	1.24	2.23	7.70	226	43.7	4.80	10.7	25.1	29.0	416	233	5
		Blended	7.07	1.18	1.82	24.6	148	33.0	3.40	11.7	18.4	25.5	514	172	7
		Acidified	5.95	0.97	0.29	17.3	125	-10.2	0.70	10.1	17.3	28.1	221	142	8
		Sieved	8.77	1.19	2.43	74.9	141	45.2	5.20	14.7	27.6	35.7	400	216	9

Supplementary Table S2. The 31 carbon substrates and corresponding plate numbers in Biolog Ecoplates classified according to type of carbon source.

Type	Plate number	Substrates
Carbohydrates	A2	β-Methyl-D-Glucoside
	B2	D-Xylose
	C2	i-Erythritol
	D2	D-Mannitol
	E2	N-Acetyl-D-Glucosamine
	G1	D-Cellobiose
	H1	α-D-Lactose
Carboxylic acids	A3	D-Galactonic Acid-γ-Lactone
	B3	D-Galacturonic Acid
	C3	2-Hydroxy Benzoic Acid
	D3	4-Hydroxy Benzoic Acid
	E3	γ-Hydroxybutyric Acid
	F2	D-Glucosaminic Acid
	F3	Itaconic Acid
	G3	α-Ketobutyric Acid
	H3	D-Malic Acid
Amino acids	A4	L-Arginine
	B4	L-Asparagine
	C4	L-Phenylalanine
	D4	L-Serine
	E4	L-Threonine
	F4	Glycyl-L-Glutamic Acid
Miscellaneous	B1	Pyruvic Acid Methyl Ester
	G2	Glucose-1-Phosphate
	H2	D,L-α-Glycerol Phosphate
Polymers	C1	Tween 40
	D1	Tween 80
	E1	α-Cyclodextrin
	F1	Glycogen
Amines/Amides	G4	Phenylethyl-amine
	H4	Putrescine

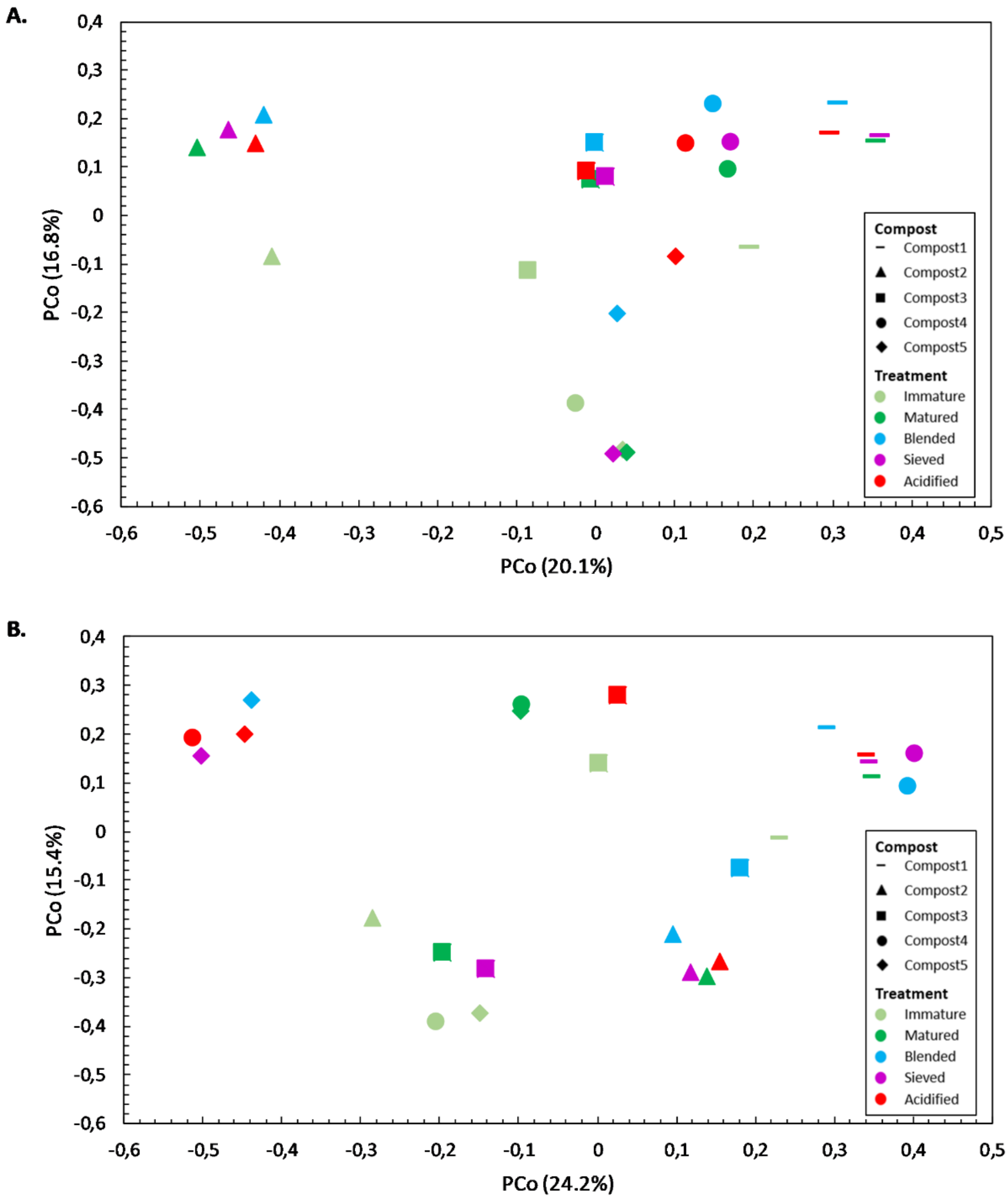

Supplementary Figure S1. Major shifts in bacterial and fungal community composition between immature, matured, blended, sieved and acidified composts. (A) Principal Coordinate Analysis (PCoA) profile of pairwise community dissimilarity (Bray-Curtis) indices of bacterial (16S V3-V4 rRNA gene) sequencing data of the treated composts. Compost batch (compost 1, compost 2, compost 3, compost 4 or compost 5) is represented by shapes. Colors indicate treatments (immature, matured, blended, sieved or acidified composts). The first and second axis represent 20.1% and 16.8%, respectively, of the variance in the dataset. A separation is seen in the first axis, representing the major amount of variance in the dataset due to compost batch. (B) PCoA profile of pairwise community dissimilarity (Bray-Curtis) indices of fungal (ITS2 gene sequencing data) of the treated composts. Compost batch (compost 1, compost 2, compost 3, compost 4 or compost 5) is represented by shapes. Colors indicate treatments (immature, matured, blended, sieved or acidified composts). The first and second axis represent 24.2% and 15.4%, respectively, of the variance in the dataset.

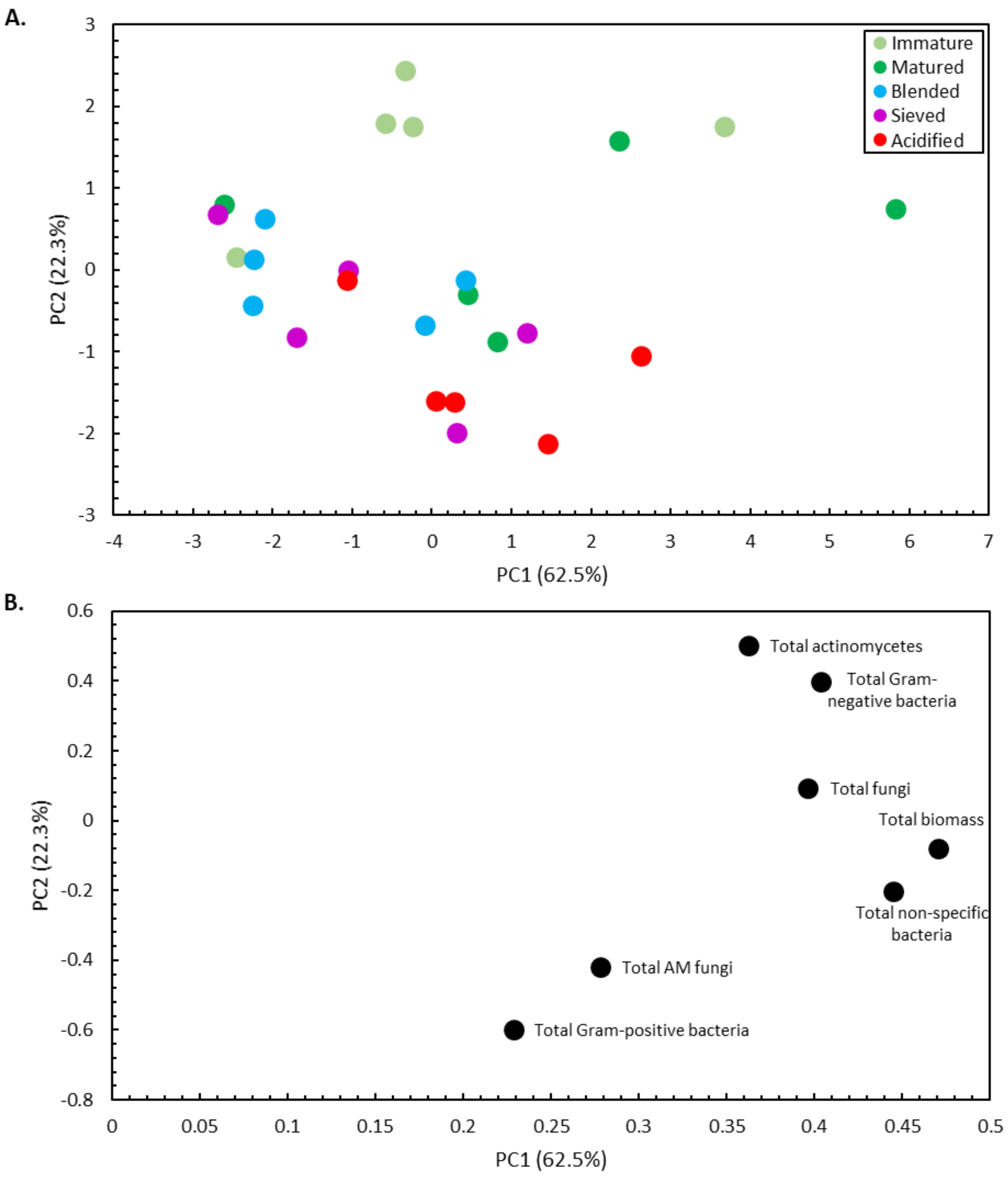

Supplementary Figure S2. Differentiation of treated composts based on the absolute abundances of microbial groups. (A) Principal component analysis (PCA) on absolute abundances of microbial groups based on PLFA analysis. Colors indicate treatment (immature, matured, blended, sieved or acidified composts). The first two principal components (PC) presented 62.5% and 22.3%, respectively, of total variability in the dataset. (B) Loadings of biomass of microbial groups and total microbial biomass on the first two principal components. PC1 was mainly determined by the absolute abundance of fungi, Gram negative bacteria, non-specific bacteria, actinomycetes and total microbial biomass. PC2 was mainly determined by the absolute abundance of arbuscular mycorrhizal fungi and Gram-positive bacteria.

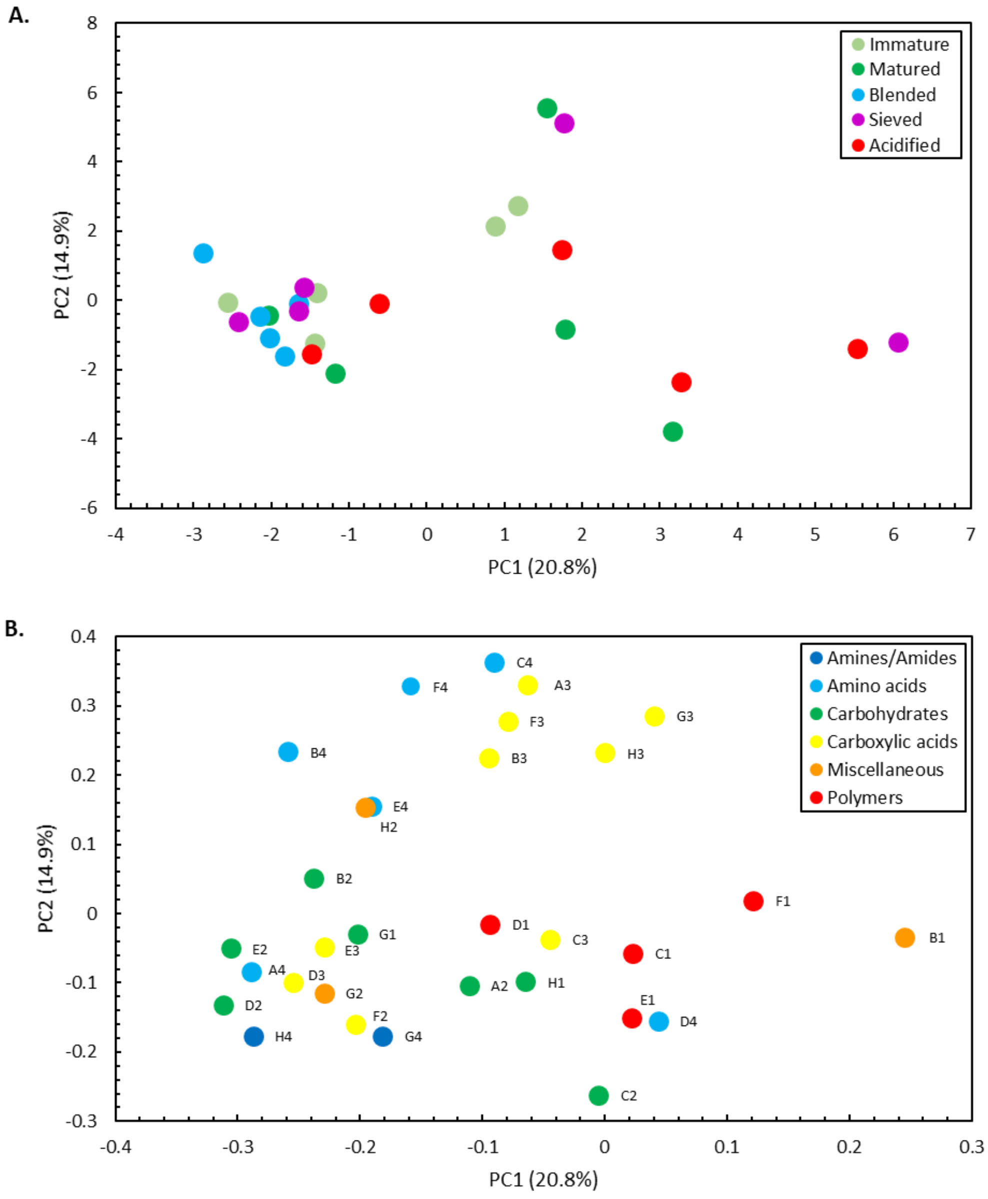

Supplementary Figure S3. Differentiation of immature, matured, blended, sieved and acidified composts based on carbon source metabolization profiles. (A) Principal component analysis (PCA) on carbon source metabolization determined with Biolog Ecoplates. Colors indicate treatment (immature, matured, blended, sieved or acidified composts). The first two principal components (PC) presented 20.79% and 14.85%, respectively, of total variability in the dataset. (B) Loadings of the 31 carbon sources in Biolog Ecoplates on the first two principal components (see Table 1 for details). The first principal component is mostly determined by pyruvic acid methyl ester and will separate groups mainly based on their ability to utilize pyruvic acid methyl ester. On the other hand, a high score on the second principal component can be related to a high utilization of a group of carboxylic acids. A lower score on the second principal component can mainly be related to the utilization of polymers, carbohydrates and amines.

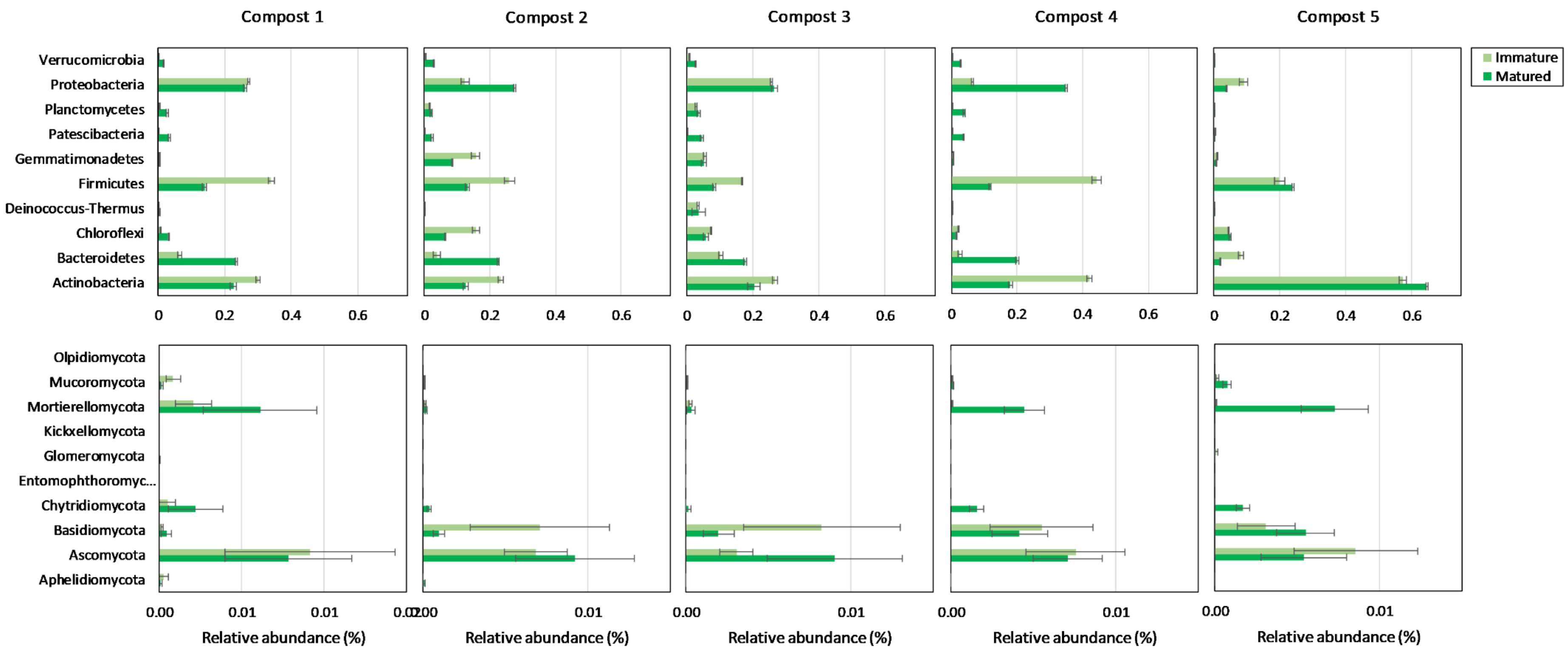

Supplementary Figure S4. Microbial composition of immature and matured composts. On top: relative abundance (percentages) ± standard error of the ten most abundant bacterial phyla (16S V3-V4 region rRNA gene) of immature and matured composts in the different compost batches. At the bottom: relative abundance (percentages) ± standard error of the ten most abundant fungal phyla (ITS2 gene) of immature and matured composts in the different compost batches.

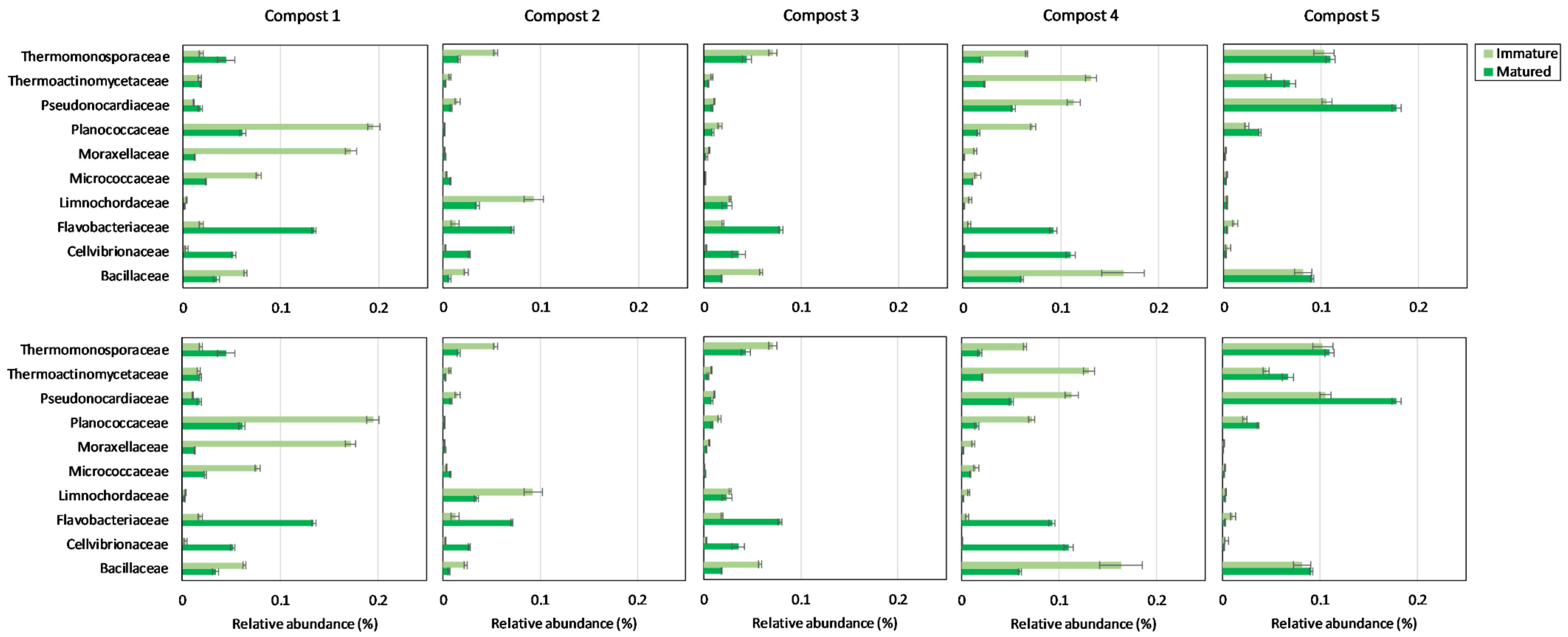

Supplementary Figure S5. Microbial composition of immature and matured composts. On top: relative abundance (percentages) ± standard error of the ten most abundant bacterial families (16S V3-V4 region rRNA gene) of immature and matured composts in the different compost batches. At the bottom: relative abundance (percentages) ± standard error of the ten most abundant fungal families (ITS2 gene) of immature and matured composts in the different compost batches.

.

Supplementary Table S3. Significantly altered bacterial phyla (16S V3-V4 region rRNA gene) due to treatment (maturation, blending, sieving or acidification) of the composts. (A) Relative abundances (percentages) ± standard error of significantly altered bacterial phyla between matured and immature composts in at least four compost batches (B) Relative abundances (percentages) ± standard error of significantly altered bacterial phyla between matured and blended composts in at least four compost batches. (C) Relative abundances (percentages) ± standard error of significantly altered bacterial phyla between matured and sieved composts in at least four compost batches. (D) Relative abundances (percentages) ± standard error of significantly altered bacterial phyla between matured and acidified composts in at least four compost batches.

A.

	Compost 1		Compost 2		Compost 3		Compost 4		Compost 5	
	Immature	Matured	Immature	Matured	Immature	Matured	Immature	Matured	Immature	Matured
Actinobacteria	3.01E-01 ± 5.14E-03	2.26E-01 ± 8.41E-03	2.33E-01 ± 7.35E-03	1.25E-01 ± 2.19E-03	2.68E-01 ± 7.57E-03	2.03E-01 ± 1.88E-02	4.20E-01 ± 8.41E-03	1.80E-01 ± 6.81E-03	5.71E-01 ± 1.14E-02	6.45E-01 ± 3.55E-03
Bacteroidetes	6.39E-02 ± 5.63E-03	2.34E-01 ± 3.00E-03	3.75E-02 ± 1.09E-02	2.26E-01 ± 9.11E-03	1.02E-01 ± 6.21E-03	1.77E-01 ± 4.24E-03	2.63E-02 ± 5.30E-03	2.01E-01 ± 4.76E-03	8.06E-02 ± 8.07E-03	1.93E-02 ± 1.58E-03
BRC1	4.07E-05 ± 4.07E-05	4.23E-03 ± 2.37E-04	2.77E-04 ± 4.77E-05	2.09E-03 ± 3.34E-04	7.51E-04 ± 1.68E-04	1.82E-03 ± 2.41E-04	2.62E-05 ± 2.62E-05	2.78E-03 ± 2.38E-04	2.90E-05 ± 2.90E-05	0.00E00 ± 0.00E00
Chlamydiae	1.75E-05 ± 1.75E-05	3.18E-04 ± 8.95E-05	8.36E-05 ± 5.76E-06	3.18E-03 ± 3.68E-04	0.00E00 ± 0.00E00	6.42E-05 ± 3.56E-05	0.00E00 ± 0.00E00	1.30E-03 ± 9.64E-05	2.62E-05 ± 2.62E-05	0.00E00 ± 0.00E00
Cyanobacteria	3.56E-05 ± 3.56E-05	4.66E-03 ± 4.97E-04	4.91E-05 ± 4.91E-05	8.48E-03 ± 3.12E-04	8.62E-04 ± 1.72E-04	5.92E-03 ± 6.00E-04	0.00E00 ± 0.00E00	3.31E-03 ± 3.11E-05	8.14E-05 ± 4.55E-05	5.60E-05 ± 5.60E-05
FBP	0.00E00 ± 0.00E00	1.40E-03 ± 2.48E-04	1.23E-05 ± 1.23E-05	1.14E-03 ± 1.41E-04	0.00E00 ± 0.00E00	4.02E-04 ± 2.42E-04	0.00E00 ± 0.00E00	2.96E-04 ± 3.75E-05	0.00E00 ± 0.00E00	0.00E00 ± 0.00E00
Firmicutes	3.40E-01 ± 9.12E-03	1.39E-01 ± 6.14E-03	2.59E-01 ± 1.52E-02	1.32E-01 ± 8.26E-03	1.68E-01 ± 2.36E-03	8.39E-02 ± 4.80E-03	4.43E-01 ± 1.49E-02	1.18E-01 ± 3.79E-03	1.99E-01 ± 1.52E-02	2.39E-01 ± 4.14E-03
Hydrogenedentes	3.56E-05 ± 3.56E-05	9.61E-04 ± 4.03E-05	0.00E00 ± 0.00E00	1.38E-03 ± 2.16E-04	2.47E-04 ± 5.02E-05	1.28E-03 ± 1.53E-04	0.00E00 ± 0.00E00	1.80E-03 ± 2.59E-04	0.00E00 ± 0.00E00	0.00E00 ± 0.00E00
Patescibacteria	3.84E-04 ± 5.80E-05	3.33E-02 ± 3.81E-03	5.24E-04 ± 3.76E-04	2.36E-02 ± 5.08E-03	1.33E-03 ± 2.06E-04	4.45E-02 ± 4.29E-03	5.00E-04 ± 3.72E-04	3.85E-02 ± 1.72E-03	2.77E-03 ± 9.91E-04	4.62E-04 ± 1.88E-04
Verrucomicrobia	2.36E-04 ± 1.24E-04	1.50E-02 ± 1.01E-03	2.74E-03 ± 1.16E-03	2.90E-02 ± 2.32E-03	6.71E-03 ± 3.89E-04	2.72E-02 ± 2.06E-03	1.02E-04 ± 2.77E-05	2.81E-02 ± 1.16E-03	5.17E-04 ± 1.97E-04	2.61E-04 ± 1.58E-04

B.

	Compost 1		Compost 2		Compost 3		Compost 4		Compost 5	
	Matured	Blended	Matured	Blended	Matured	Blended	Matured	Blended	Matured	Blended
Firmicutes	1.39E-01 ± 6.14E-03	1.02E-01 ± 2.33E-03	1.32E-01 ± 8.26E-03	1.03E-01 ± 1.13E-02	8.39E-02 ± 4.80E-03	7.79E-02 ± 4.19E-03	1.18E-01 ± 3.79E-03	8.77E-02 ± 4.74E-03	2.39E-01 ± 4.14E-03	1.14E-01 ± 1.01E-03
Gemmatimonadetes	2.89E-03 ± 2.63E-04	5.96E-03 ± 2.68E-04	8.45E-02 ± 2.62E-03	6.54E-02 ± 1.89E-03	5.31E-02 ± 8.02E-03	3.76E-02 ± 1.52E-03	5.94E-03 ± 1.04E-03	4.21E-03 ± 4.54E-04	6.11E-03 ± 3.73E-04	3.83E-03 ± 2.24E-04

C.

	Compost 1		Compost 2		Compost 3		Compost 4		Compost 5	
	Matured	Sieved	Matured	Sieved	Matured	Sieved	Matured	Sieved	Matured	Sieved
Cyanobacteria	4.66E-03 ± 4.97E-04	2.04E-03 ± 1.81E-04	8.48E-03 ± 3.12E-04	4.14E-03 ± 4.78E-04	5.92E-03 ± 6.00E-04	1.77E-03 ± 2.24E-04	3.31E-03 ± 3.11E-05	1.67E-03 ± 1.55E-04	5.60E-05 ± 5.60E-05	3.23E-05 ± 3.23E-05
Deinococcus-Thermus	3.13E-03 ± 3.91E-04	1.03E-02 ± 9.32E-04	8.34E-05 ± 4.21E-05	5.13E-04 ± 7.28E-05	3.70E-02 ± 2.03E-02	8.90E-03 ± 1.12E-03	9.19E-04 ± 2.17E-04	2.73E-03 ± 1.45E-04	4.48E-04 ± 1.77E-04	8.31E-04 ± 5.38E-04

D.

	Compost 1		Compost 2		Compost 3		Compost 4		Compost 5	
	Matured	Acidified	Matured	Acidified	Matured	Acidified	Matured	Acidified	Matured	Acidified
Proteobacteria	2.61E-01 ± 3.93E-03	4.19E-01 ± 6.39E-03	2.75E-01 ± 7.82E-03	5.27E-01 ± 1.90E-02	2.64E-01 ± 8.87E-03	4.43E-01 ± 1.42E-02	3.49E-01 ± 2.73E-03	5.25E-01 ± 1.89E-02	3.82E-02 ± 1.78E-03	4.29E-01 ± 5.31E-03

Supplementary Table S4. Significantly altered bacterial families (16S V3-V4 region rRNA gene) due to treatment (maturation, blending, sieving or acidification) of the composts. (A) Relative abundances (percentages) ± standard error of significantly altered bacterial families between matured and immature composts in at least four compost batches (B) Relative abundances (percentages) ± standard error of significantly altered bacterial families between matured and blended composts in at least four compost batches. (C) Relative abundances (percentages) ± standard error of significantly altered bacterial families between matured and sieved composts in at least four compost batches. (D) Relative abundances (percentages) ± standard error of significantly altered bacterial families between matured and acidified composts in at least four compost batches.

A.

	Compost 1		Compost 2		Compost 3		Compost 4		Compost 5	
	Immature	Matured	Immature	Matured	Immature	Matured	Immature	Matured	Immature	Matured
Archangiaceae	0.00E00 ± 0.00E00	2.20E-04 ± 2.20E-04	0.00E00 ± 0.00E00	2.80E-03 ± 1.12E-04	2.25E-04 ± 1.02E-05	1.12E-03 ± 1.73E-04	0.00E00 ± 0.00E00	5.43E-04 ± 1.06E-05	0.00E00 ± 0.00E00	0.00E00 ± 0.00E00
Bacillaceae	6.39E-02 ± 1.55E-03	3.45E-02 ± 3.17E-03	2.36E-02 ± 1.98E-03	6.77E-03 ± 1.10E-03	5.84E-02 ± 1.59E-03	1.86E-02 ± 4.78E-04	1.64E-01 ± 2.18E-02	6.02E-02 ± 1.84E-03	8.17E-02 ± 8.84E-03	9.09E-02 ± 1.66E-03
Bacteriovoracaceae	0.00E00 ± 0.00E00	2.90E-04 ± 9.35E-05	3.75E-05 ± 3.75E-05	7.10E-04 ± 2.66E-04	1.75E-04 ± 4.51E-05	1.72E-03 ± 1.66E-04	8.47E-05 ± 8.47E-05	2.02E-03 ± 2.99E-05	0.00E00 ± 0.00E00	0.00E00 ± 0.00E00
Burkholderiaceae	8.90E-03 ± 8.94E-04	2.14E-02 ± 1.40E-03	1.20E-02 ± 3.67E-03	4.62E-02 ± 5.59E-03	1.90E-02 ± 1.25E-03	3.13E-02 ± 1.18E-03	1.09E-02 ± 9.64E-04	3.24E-02 ± 1.55E-03	2.00E-02 ± 1.88E-03	7.41E-03 ± 8.25E-04
Caulobacteraceae	0.00E00 ± 0.00E00	2.20E-04 ± 1.11E-04	5.08E-04 ± 8.73E-05	7.29E-03 ± 5.43E-04	3.98E-04 ± 6.23E-05	1.12E-03 ± 3.42E-04	2.61E-05 ± 2.61E-05	3.23E-03 ± 4.11E-04	2.90E-05 ± 2.90E-05	0.00E00 ± 0.00E00
Cellvibrionaceae	3.83E-03 ± 1.41E-03	5.18E-02 ± 1.84E-03	2.15E-03 ± 3.99E-04	2.74E-02 ± 1.14E-03	1.92E-03 ± 1.03E-04	3.57E-02 ± 6.76E-03	6.93E-04 ± 3.14E-04	1.10E-01 ± 4.54E-03	4.18E-03 ± 2.18E-03	1.95E-03 ± 5.14E-04
Chthoniobacteraceae	0.00E00 ± 0.00E00	1.06E-03 ± 9.09E-05	3.07E-05 ± 3.07E-05	3.04E-03 ± 4.02E-04	1.05E-03 ± 9.12E-05	3.67E-03 ± 6.78E-04	0.00E00 ± 0.00E00	2.85E-03 ± 1.68E-04	0.00E00 ± 0.00E00	4.80E-05 ± 4.80E-05
Cryomorphaceae	1.41E-03 ± 2.45E-04	1.54E-02 ± 7.66E-04	3.12E-05 ± 3.12E-05	1.16E-04 ± 2.11E-05	3.40E-04 ± 2.35E-04	6.82E-03 ± 5.38E-04	0.00E00 ± 0.00E00	1.63E-02 ± 3.58E-04	0.00E00 ± 0.00E00	0.00E00 ± 0.00E00
Cyclobacteriaceae	3.27E-03 ± 5.79E-04	1.91E-02 ± 1.19E-03	2.94E-04 ± 6.42E-05	1.66E-02 ± 1.30E-03	3.55E-03 ± 3.97E-04	1.29E-02 ± 1.83E-04	1.94E-04 ± 2.93E-05	1.64E-02 ± 1.19E-03	1.88E-03 ± 9.61E-04	2.21E-04 ± 1.48E-04
Devosiaceae	2.65E-03 ± 3.59E-04	1.87E-02 ± 1.78E-03	3.68E-03 ± 8.79E-05	3.33E-02 ± 2.63E-04	4.52E-04 ± 1.36E-04	9.90E-03 ± 1.08E-03	7.92E-04 ± 1.18E-04	2.48E-02 ± 8.90E-04	2.16E-03 ± 1.78E-04	6.19E-04 ± 2.47E-04
Flavobacteriaceae	1.88E-02 ± 1.92E-03	1.34E-01 ± 2.10E-03	1.28E-02 ± 4.24E-03	7.12E-02 ± 1.20E-03	1.92E-02 ± 1.06E-03	7.88E-02 ± 2.06E-03	6.10E-03 ± 1.50E-03	9.27E-02 ± 3.36E-03	1.15E-02 ± 2.61E-03	2.60E-03 ± 1.15E-04
Hydrogenedensaceae	3.56E-05 ± 3.56E-05	9.61E-04 ± 4.03E-05	0.00E00 ± 0.00E00	1.38E-03 ± 2.16E-04	2.47E-04 ± 5.02E-05	1.28E-03 ± 1.53E-04	0.00E00 ± 0.00E00	1.80E-03 ± 2.59E-04	0.00E00 ± 0.00E00	0.00E00 ± 0.00E00
Methylophagaceae	9.32E-04 ± 5.09E-05	4.75E-03 ± 8.49E-04	0.00E00 ± 0.00E00	3.07E-04 ± 1.14E-04	0.00E00 ± 0.00E00	1.01E-03 ± 4.78E-04	4.46E-04 ± 1.60E-04	4.45E-03 ± 6.41E-04	5.66E-03 ± 1.93E-03	3.23E-03 ± 4.94E-04
Micavibrionaceae	0.00E00 ± 0.00E00	1.68E-04 ± 3.98E-05	0.00E00 ± 0.00E00	3.33E-04 ± 1.86E-05	0.00E00 ± 0.00E00	1.38E-03 ± 2.80E-04	0.00E00 ± 0.00E00	7.36E-05 ± 3.74E-05	0.00E00 ± 0.00E00	0.00E00 ± 0.00E00
Microbacteriaceae	2.65E-03 ± 1.44E-04	1.43E-02 ± 7.68E-04	1.61E-03 ± 9.40E-05	9.90E-03 ± 9.67E-04	5.29E-04 ± 2.60E-04	5.36E-03 ± 3.13E-04	4.67E-03 ± 3.18E-04	1.55E-02 ± 2.01E-03	1.60E-02 ± 6.26E-04	8.07E-03 ± 1.15E-03
Micropepsaceae	0.00E00 ± 0.00E00	3.83E-04 ± 5.48E-05	0.00E00 ± 0.00E00	6.64E-04 ± 5.42E-05	9.82E-05 ± 9.82E-05	4.39E-04 ± 9.77E-05	0.00E00 ± 0.00E00	9.30E-04 ± 1.19E-05	0.00E00 ± 0.00E00	1.92E-04 ± 1.92E-04
NS9_marine_group	1.19E-05 ± 1.19E-05	1.62E-03 ± 3.01E-04	6.13E-05 ± 6.13E-05	2.23E-04 ± 2.70E-05	4.72E-04 ± 7.43E-05	1.85E-03 ± 1.09E-04	0.00E00 ± 0.00E00	1.77E-03 ± 3.20E-04	0.00E00 ± 0.00E00	0.00E00 ± 0.00E00
Oligoflexaceae	0.00E00 ± 0.00E00	2.35E-03 ± 1.71E-04	0.00E00 ± 0.00E00	7.61E-05 ± 4.05E-05	9.74E-05 ± 5.09E-05	1.37E-03 ± 1.82E-04	0.00E00 ± 0.00E00	9.94E-04 ± 1.82E-04	0.00E00 ± 0.00E00	0.00E00 ± 0.00E00
Opitutaceae	8.57E-05 ± 4.50E-05	6.65E-04 ± 6.82E-05	2.23E-05 ± 2.23E-05	4.88E-03 ± 6.33E-04	4.68E-04 ± 1.62E-05	1.77E-03 ± 2.99E-04	4.06E-05 ± 4.06E-05	4.67E-03 ± 1.83E-04	0.00E00 ± 0.00E00	0.00E00 ± 0.00E00
Parachlamydiaceae	0.00E00 ± 0.00E00	3.18E-04 ± 8.95E-05	3.12E-05 ± 3.12E-05	3.18E-03 ± 3.68E-04	0.00E00 ± 0.00E00	5.05E-05 ± 2.55E-05	0.00E00 ± 0.00E00	5.60E-04 ± 8.67E-05	0.00E00 ± 0.00E00	0.00E00 ± 0.00E00
Rubritaleaceae	0.00E00 ± 0.00E00	1.58E-03 ± 2.99E-04	9.47E-04 ± 2.98E-04	5.55E-03 ± 5.49E-04	4.40E-04 ± 2.12E-05	6.77E-03 ± 1.16E-03	0.00E00 ± 0.00E00	7.30E-03 ± 4.47E-04	1.92E-04 ± 9.71E-05	3.80E-05 ± 3.80E-05
Saccharimonadaceae	3.43E-04 ± 4.01E-05	1.12E-02 ± 1.44E-03	3.63E-04 ± 2.83E-04	5.76E-03 ± 8.58E-04	2.89E-04 ± 5.45E-05	8.79E-03 ± 6.79E-04	3.26E-05 ± 3.26E-05	9.92E-03 ± 5.93E-04	2.34E-03 ± 9.10E-04	4.62E-04 ± 1.88E-04
Saprospiraceae	1.06E-03 ± 5.22E-05	8.44E-03 ± 1.34E-04	0.00E00 ± 0.00E00	1.45E-03 ± 9.65E-05	4.72E-04 ± 6.74E-05	4.19E-03 ± 4.17E-04	0.00E00 ± 0.00E00	1.25E-02 ± 8.54E-04	2.05E-05 ± 2.05E-05	3.20E-05 ± 3.20E-05
Sphingomonadaceae	8.43E-04 ± 8.01E-05	6.49E-03 ± 6.67E-04	3.60E-03 ± 6.50E-04	1.38E-02 ± 1.13E-03	3.68E-03 ± 4.49E-04	7.48E-03 ± 2.01E-04	2.61E-04 ± 1.38E-04	1.17E-02 ± 6.69E-04	1.99E-03 ± 2.29E-04	6.06E-04 ± 8.30E-05
Xanthomonadaceae	4.34E-04 ± 4.63E-05	1.02E-02 ± 9.95E-04	3.26E-03 ± 2.64E-04	1.13E-02 ± 3.39E-04	1.86E-02 ± 3.90E-04	2.51E-02 ± 1.02E-03	1.22E-03 ± 2.38E-04	1.40E-02 ± 8.09E-04	3.73E-04 ± 9.27E-05	2.32E-05 ± 2.32E-05

B.

	Compost 1		Compost 2		Compost 3		Compost 4		Compost 5	
	Matured	Blended	Matured	Blended	Matured	Blended	Matured	Blended	Matured	Blended
67-14	4.42E-04 ± 6.30E-05	2.70E-03 ± 4.46E-04	9.01E-05 ± 9.01E-05	5.04E-03 ± 5.22E-04	9.13E-04 ± 1.70E-04	2.62E-03 ± 2.25E-04	4.12E-04 ± 9.06E-06	2.52E-03 ± 3.06E-04	6.90E-04 ± 2.19E-04	1.13E-03 ± 2.51E-04
Acetobacteraceae	0.00E00 ± 0.00E00	8.73E-03 ± 2.94E-04	6.38E-04 ± 1.95E-04	4.98E-03 ± 6.62E-04	2.34E-04 ± 1.60E-04	2.89E-03 ± 2.74E-04	8.99E-05 ± 4.96E-05	6.44E-03 ± 2.33E-04	8.55E-05 ± 8.11E-06	1.17E-02 ± 4.35E-04
Acidobacteriaceae_(Subgroup_1)	0.00E00 ± 0.00E00	1.60E-02 ± 1.72E-03	0.00E00 ± 0.00E00	2.29E-02 ± 2.82E-03	0.00E00 ± 0.00E00	1.49E-02 ± 1.65E-03	0.00E00 ± 0.00E00	1.13E-02 ± 7.77E-04	6.40E-05 ± 6.40E-05	1.81E-02 ± 9.82E-04
Acidothermaceae	0.00E00 ± 0.00E00	5.39E-03 ± 5.83E-04	0.00E00 ± 0.00E00	3.34E-03 ± 4.34E-04	0.00E00 ± 0.00E00	3.07E-03 ± 1.31E-03	0.00E00 ± 0.00E00	4.33E-03 ± 5.64E-04	0.00E00 ± 0.00E00	3.99E-03 ± 1.36E-04
Beijerinckiaceae	1.56E-04 ± 1.56E-04	4.50E-03 ± 2.21E-04	2.35E-03 ± 2.27E-04	7.92E-03 ± 4.30E-04	1.66E-03 ± 2.00E-04	6.15E-03 ± 3.61E-04	7.98E-04 ± 2.44E-05	4.40E-03 ± 4.51E-04	2.30E-04 ± 1.87E-04	3.85E-03 ± 3.46E-04
Chitinophagaceae	2.85E-03 ± 2.22E-04	1.21E-02 ± 1.25E-03	1.40E-02 ± 1.17E-03	3.47E-02 ± 7.19E-04	6.77E-03 ± 5.12E-04	2.33E-02 ± 7.10E-04	3.08E-03 ± 3.39E-04	1.32E-02 ± 1.55E-03	2.54E-04 ± 1.99E-04	8.52E-03 ± 7.00E-04
CPla-3_termite_group	0.00E00 ± 0.00E00	2.99E-03 ± 2.04E-04	0.00E00 ± 0.00E00	4.12E-03 ± 2.24E-04	0.00E00 ± 0.00E00	1.83E-03 ± 6.26E-05	0.00E00 ± 0.00E00	2.24E-03 ± 3.28E-04	0.00E00 ± 0.00E00	4.05E-03 ± 2.42E-04
Crocinitomicaceae	8.23E-03 ± 8.36E-04	9.93E-04 ± 2.06E-04	2.61E-02 ± 9.93E-04	4.79E-03 ± 2.42E-04	9.29E-03 ± 1.63E-03	3.06E-03 ± 3.84E-04	1.19E-02 ± 7.37E-04	5.34E-03 ± 3.44E-04	2.88E-04 ± 2.44E-05	2.02E-04 ± 6.68E-05
Gemmataceae	0.00E00 ± 0.00E00	2.54E-03 ± 5.37E-05	0.00E00 ± 0.00E00	9.47E-04 ± 4.63E-04	6.97E-05 ± 6.97E-05	6.04E-04 ± 1.73E-04	0.00E00 ± 0.00E00	1.66E-03 ± 2.71E-04	6.40E-05 ± 6.40E-05	2.78E-03 ± 2.69E-04
Gemmatimonadaceae	0.00E00 ± 0.00E00	3.54E-03 ± 8.32E-05	1.43E-03 ± 2.33E-04	8.70E-03 ± 3.86E-04	1.12E-03 ± 1.50E-04	6.01E-03 ± 3.20E-04	9.27E-04 ± 2.26E-04	4.46E-03 ± 3.97E-04	0.00E00 ± 0.00E00	5.44E-04 ± 3.23E-05
Isosphaeraceae	0.00E00 ± 0.00E00	1.21E-03 ± 2.50E-04	2.00E-04 ± 4.68E-05	1.93E-03 ± 4.53E-04	1.17E-03 ± 1.89E-04	1.92E-03 ± 4.04E-04	0.00E00 ± 0.00E00	8.80E-04 ± 1.97E-04	0.00E00 ± 0.00E00	6.69E-04 ± 2.72E-05
JG30-KF-AS9	0.00E00 ± 0.00E00	2.46E-02 ± 1.76E-03	0.00E00 ± 0.00E00	1.37E-02 ± 7.47E-04	0.00E00 ± 0.00E00	9.51E-03 ± 1.63E-03	0.00E00 ± 0.00E00	1.70E-02 ± 2.56E-03	0.00E00 ± 0.00E00	2.27E-02 ± 2.76E-03
Ktedonobacteraceae	0.00E00 ± 0.00E00	4.74E-04 ± 7.56E-05	0.00E00 ± 0.00E00	1.65E-03 ± 2.21E-05	0.00E00 ± 0.00E00	1.37E-03 ± 1.61E-04	0.00E00 ± 0.00E00	3.52E-04 ± 2.56E-05	0.00E00 ± 0.00E00	9.75E-05 ± 5.20E-05
Micrococcaceae	2.35E-02 ± 9.78E-04	8.11E-03 ± 1.47E-03	7.89E-03 ± 5.76E-04	4.85E-03 ± 2.45E-04	1.49E-03 ± 1.46E-04	5.78E-04 ± 7.27E-05	8.88E-03 ± 2.52E-04	3.89E-03 ± 4.61E-04	2.11E-03 ± 4.04E-04	4.75E-02 ± 1.31E-03
Micropepsaceae	3.83E-04 ± 5.48E-05	6.32E-03 ± 8.66E-04	6.64E-04 ± 5.42E-05	2.70E-02 ± 3.79E-03	4.39E-04 ± 9.77E-05	1.06E-02 ± 1.30E-03	9.30E-04 ± 1.19E-05	6.44E-03 ± 3.23E-04	1.92E-04 ± 1.92E-04	5.92E-03 ± 7.53E-04
Mitochondria	7.76E-04 ± 5.84E-05	1.37E-03 ± 1.92E-04	0.00E00 ± 0.00E00	3.48E-03 ± 5.70E-04	0.00E00 ± 0.00E00	2.30E-03 ± 2.50E-04	8.20E-05 ± 5.00E-05	2.16E-03 ± 6.73E-04	0.00E00 ± 0.00E00	1.34E-03 ± 1.69E-04
Opitutaceae	6.65E-04 ± 6.82E-05	2.18E-03 ± 1.61E-04	4.88E-03 ± 6.33E-04	7.13E-03 ± 1.42E-03	1.77E-03 ± 2.99E-04	3.65E-03 ± 2.26E-04	4.67E-03 ± 1.83E-04	6.90E-03 ± 1.84E-03	0.00E00 ± 0.00E00	2.98E-04 ± 1.56E-04
Pedosphaeraceae	0.00E00 ± 0.00E00	7.32E-03 ± 4.02E-04	6.04E-05 ± 3.97E-05	1.55E-02 ± 1.44E-03	2.38E-04 ± 2.06E-05	4.43E-03 ± 1.69E-04	0.00E00 ± 0.00E00	7.39E-03 ± 4.59E-04	1.20E-04 ± 1.20E-04	1.33E-02 ± 1.41E-03
Rhodanobacteraceae	2.75E-04 ± 6.72E-05	3.28E-02 ± 6.43E-04	6.72E-04 ± 5.85E-05	6.04E-02 ± 3.57E-03	6.50E-04 ± 4.48E-05	2.14E-02 ± 6.71E-04	6.97E-04 ± 4.13E-04	2.36E-02 ± 1.50E-03	0.00E00 ± 0.00E00	4.36E-02 ± 1.45E-03
Solibacteraceae_(Subgroup_3)	8.87E-05 ± 1.81E-05	8.87E-04 ± 1.36E-04	1.15E-04 ± 6.51E-05	6.25E-03 ± 6.68E-04	0.00E00 ± 0.00E00	4.22E-03 ± 3.88E-04	0.00E00 ± 0.00E00	6.80E-04 ± 4.77E-05	0.00E00 ± 0.00E00	3.50E-05 ± 3.50E-05
Solirubrobacteraceae	1.78E-03 ± 1.78E-04	8.41E-03 ± 1.53E-03	0.00E00 ± 0.00E00	4.42E-03 ± 1.14E-03	7.04E-05 ± 7.04E-05	3.08E-03 ± 1.45E-04	0.00E00 ± 0.00E00	2.65E-03 ± 2.63E-04	9.18E-05 ± 4.76E-05	2.72E-03 ± 3.42E-04
Sporichthyaceae	0.00E00 ± 0.00E00	1.25E-03 ± 9.19E-05	0.00E00 ± 0.00E00	1.03E-03 ± 1.36E-04	0.00E00 ± 0.00E00	3.71E-04 ± 1.88E-04	0.00E00 ± 0.00E00	1.13E-03 ± 4.84E-05	0.00E00 ± 0.00E00	1.22E-03 ± 6.97E-05
Unknown_Family	2.31E-03 ± 1.14E-03	1.44E-02 ± 6.48E-03	0.00E00 ± 0.00E00	6.62E-03 ± 3.89E-03	3.51E-04 ± 4.27E-05	5.38E-03 ± 2.21E-03	1.23E-04 ± 5.94E-05	8.62E-03 ± 3.94E-03	3.20E-05 ± 3.20E-05	1.73E-02 ± 7.74E-03
WD2101_soil_group	1.29E-03 ± 2.49E-04	4.58E-03 ± 4.01E-04	1.31E-04 ± 7.19E-05	6.29E-03 ± 4.72E-04	2.49E-03 ± 5.29E-04	8.04E-03 ± 9.72E-04	1.79E-03 ± 3.80E-04	6.08E-03 ± 3.33E-04	1.60E-05 ± 1.60E-05	4.29E-03 ± 4.28E-04
Xanthobacteraceae	5.40E-04 ± 5.44E-05	1.27E-02 ± 1.01E-03	5.83E-03 ± 2.08E-04	2.52E-02 ± 7.61E-04	5.09E-04 ± 4.53E-05	1.33E-02 ± 2.76E-04	2.40E-03 ± 2.19E-04	1.23E-02 ± 1.97E-04	2.80E-04 ± 2.80E-04	9.76E-03 ± 1.11E-03

C.

	Compost 1		Compost 2		Compost 3		Compost 4		Compost 5	
	Matured	Sieved	Matured	Sieved	Matured	Sieved	Matured	Sieved	Matured	Sieved
Blrii41	1.29E-03 ± 2.49E-04	2.33E-03 ± 2.45E-04	3.75E-05 ± 3.75E-05	1.46E-02 ± 2.44E-03	3.29E-04 ± 8.53E-05	1.36E-02 ± 1.78E-04	2.50E-03 ± 5.83E-04	9.35E-03 ± 3.46E-04	1.12E-04 ± 1.12E-04	6.23E-05 ± 6.23E-05
Crocinitomicaceae	2.32E-02 ± 3.17E-04	2.01E-03 ± 3.78E-04	2.61E-02 ± 9.93E-04	9.12E-03 ± 5.80E-04	9.29E-03 ± 1.63E-03	5.18E-03 ± 7.68E-04	1.19E-02 ± 7.37E-04	4.18E-03 ± 3.33E-04	2.88E-04 ± 2.44E-05	0.00E00 ± 0.00E00
Isosphaeraceae	2.75E-04 ± 6.72E-05	5.04E-04 ± 2.27E-04	2.00E-04 ± 4.68E-05	1.27E-03 ± 9.75E-05	1.17E-03 ± 1.89E-04	2.85E-03 ± 1.20E-04	0.00E00 ± 0.00E00	1.82E-04 ± 9.30E-05	0.00E00 ± 0.00E00	3.23E-05 ± 3.23E-05
Rhodanobacteraceae	0.00E00 ± 0.00E00	6.06E-04 ± 2.07E-04	6.72E-04 ± 5.85E-05	6.46E-03 ± 1.76E-03	6.50E-04 ± 4.48E-05	7.28E-03 ± 6.82E-04	6.97E-04 ± 4.13E-04	3.66E-03 ± 1.31E-04	0.00E00 ± 0.00E00	4.33E-04 ± 2.64E-04
WD2101_soil_group	3.89E-05 ± 3.89E-05	2.68E-03 ± 3.11E-04	1.31E-04 ± 7.19E-05	5.50E-03 ± 3.33E-05	2.49E-03 ± 5.29E-04	5.21E-03 ± 4.23E-04	1.79E-03 ± 3.80E-04	6.90E-03 ± 5.20E-04	1.60E-05 ± 1.60E-05	8.87E-05 ± 8.87E-05

D.

	Compost 1		Compost 2		Compost 3		Compost 4		Compost 5	
	Matured	Acidified	Matured	Acidified	Matured	Acidified	Matured	Acidified	Matured	Acidified
Acholeplasmataceae	2.53E-03 ± 4.21E-04	4.77E-04 ± 8.10E-05	4.91E-04 ± 1.17E-04	3.52E-04 ± 9.72E-05	1.30E-03 ± 2.43E-04	0.00E00 ± 0.00E00	7.94E-03 ± 5.68E-04	1.14E-03 ± 1.44E-04	0.00E00 ± 0.00E00	9.60E-04 ± 1.63E-04
Alcanivoracaceae	6.08E-03 ± 9.65E-04	2.59E-02 ± 4.31E-04	0.00E00 ± 0.00E00	1.09E-03 ± 3.59E-04	4.34E-04 ± 1.29E-04	2.04E-02 ± 2.33E-03	2.79E-04 ± 2.21E-04	5.92E-03 ± 3.27E-04	1.47E-04 ± 7.56E-05	1.47E-03 ± 2.39E-04
Alteromonadaceae	1.16E-03 ± 4.99E-04	2.37E-04 ± 1.36E-04	7.21E-03 ± 8.26E-04	0.00E00 ± 0.00E00	5.82E-03 ± 1.56E-03	9.93E-04 ± 3.03E-04	4.63E-03 ± 5.52E-04	1.02E-03 ± 1.88E-04	0.00E00 ± 0.00E00	6.44E-04 ± 1.88E-04
Burkholderiaceae	2.14E-02 ± 1.40E-03	1.95E-02 ± 8.47E-04	4.62E-02 ± 5.59E-03	2.04E-02 ± 1.22E-03	3.13E-02 ± 1.18E-03	1.84E-02 ± 7.32E-04	3.24E-02 ± 1.55E-03	3.05E-02 ± 1.70E-03	7.41E-03 ± 8.25E-04	6.26E-02 ± 2.54E-03
Chitinophagaceae	2.85E-03 ± 2.22E-04	1.21E-02 ± 9.38E-05	1.40E-02 ± 1.17E-03	3.18E-02 ± 8.83E-04	6.77E-03 ± 5.12E-04	3.18E-02 ± 3.51E-03	3.08E-03 ± 3.39E-04	5.08E-03 ± 2.16E-04	2.54E-04 ± 1.99E-04	3.93E-03 ± 2.10E-04
Cryomorphaceae	1.54E-02 ± 7.66E-04	4.03E-03 ± 3.77E-04	1.16E-04 ± 2.11E-05	0.00E00 ± 0.00E00	6.82E-03 ± 5.38E-04	3.09E-04 ± 3.62E-05	1.63E-02 ± 3.58E-04	7.95E-03 ± 5.26E-04	0.00E00 ± 0.00E00	1.80E-03 ± 6.45E-05
Cyclobacteriaceae	1.91E-02 ± 1.19E-03	8.01E-03 ± 8.17E-04	1.66E-02 ± 1.30E-03	4.43E-03 ± 5.18E-04	1.29E-02 ± 1.83E-04	2.62E-03 ± 2.05E-04	1.64E-02 ± 1.19E-03	7.63E-03 ± 7.89E-04	2.21E-04 ± 1.48E-04	2.91E-02 ± 7.03E-04
D05-2	4.00E-03 ± 7.77E-05	8.65E-03 ± 6.41E-05	0.00E00 ± 0.00E00	9.00E-04 ± 2.33E-04	4.71E-04 ± 4.46E-05	7.45E-03 ± 6.94E-04	1.15E-02 ± 1.72E-03	2.27E-02 ± 5.96E-04	0.00E00 ± 0.00E00	2.28E-02 ± 3.62E-04
Ectothiorhodospiraceae	0.00E00 ± 0.00E00	2.76E-02 ± 1.15E-03	0.00E00 ± 0.00E00	0.00E00 ± 0.00E00	0.00E00 ± 0.00E00	9.55E-03 ± 1.29E-03	0.00E00 ± 0.00E00	9.45E-03 ± 2.04E-04	0.00E00 ± 0.00E00	2.98E-02 ± 1.51E-03
Fibrobacteraceae	2.01E-04 ± 3.90E-05	0.00E00 ± 0.00E00	8.88E-05 ± 8.88E-05	0.00E00 ± 0.00E00	1.55E-03 ± 3.69E-04	0.00E00 ± 0.00E00	1.42E-03 ± 6.93E-05	1.09E-04 ± 8.33E-05	0.00E00 ± 0.00E00	0.00E00 ± 0.00E00
Gemmatimonadaceae	0.00E00 ± 0.00E00	2.39E-03 ± 2.56E-04	1.43E-03 ± 2.33E-04	4.82E-03 ± 3.32E-04	1.12E-03 ± 1.50E-04	2.66E-03 ± 3.34E-04	9.27E-04 ± 2.26E-04	1.45E-03 ± 2.07E-04	0.00E00 ± 0.00E00	6.61E-05 ± 6.61E-05
Halothiobacillaceae	0.00E00 ± 0.00E00	0.00E00 ± 0.00E00	0.00E00 ± 0.00E00	1.27E-04 ± 6.61E-05	0.00E00 ± 0.00E00	7.42E-03 ± 6.70E-04	0.00E00 ± 0.00E00	2.91E-02 ± 1.79E-03	0.00E00 ± 0.00E00	3.39E-03 ± 4.50E-04
Hydrogenophilaceae	0.00E00 ± 0.00E00	1.50E-01 ± 6.36E-03	0.00E00 ± 0.00E00	2.38E-01 ± 1.05E-02	0.00E00 ± 0.00E00	9.54E-02 ± 4.93E-03	0.00E00 ± 0.00E00	1.45E-01 ± 6.01E-03	0.00E00 ± 0.00E00	4.25E-02 ± 2.74E-04
Iamiaceae	1.30E-03 ± 2.74E-04	3.53E-03 ± 2.95E-04	1.92E-03 ± 2.53E-04	2.49E-04 ± 1.25E-04	9.72E-04 ± 1.47E-04	1.81E-03 ± 1.07E-04	5.06E-03 ± 5.44E-04	7.44E-03 ± 4.24E-04	3.80E-05 ± 3.80E-05	2.82E-04 ± 2.76E-05
Isosphaeraceae	0.00E00 ± 0.00E00	2.86E-03 ± 4.14E-04	2.00E-04 ± 4.68E-05	1.92E-03 ± 6.03E-04	1.17E-03 ± 1.89E-04	3.10E-03 ± 2.80E-04	0.00E00 ± 0.00E00	0.00E00 ± 0.00E00	0.00E00 ± 0.00E00	4.74E-04 ± 1.17E-04
NS9_marine_group	1.62E-03 ± 3.01E-04	4.71E-03 ± 6.78E-04	2.23E-04 ± 2.70E-05	1.45E-03 ± 2.91E-04	1.85E-03 ± 1.09E-04	4.91E-03 ± 1.60E-04	1.77E-03 ± 3.20E-04	4.36E-03 ± 3.47E-04	0.00E00 ± 0.00E00	0.00E00 ± 0.00E00
Parvibaculaceae	1.78E-03 ± 3.54E-04	1.04E-02 ± 1.79E-04	1.52E-03 ± 1.41E-04	9.22E-03 ± 8.85E-04	7.04E-04 ± 1.36E-04	7.15E-03 ± 1.57E-03	4.34E-03 ± 2.21E-04	2.35E-03 ± 4.64E-04	0.00E00 ± 0.00E00	1.88E-03 ± 4.06E-04
Porticoccaceae	1.50E-03 ± 1.74E-04	3.07E-04 ± 1.74E-04	2.15E-03 ± 3.18E-04	6.06E-04 ± 3.70E-04	2.99E-03 ± 3.63E-04	1.75E-04 ± 1.75E-04	2.56E-03 ± 2.24E-04	6.73E-04 ± 8.60E-05	5.36E-04 ± 8.41E-05	6.09E-03 ± 1.13E-03
Pseudohongiellaceae	3.73E-03 ± 3.93E-04	6.18E-04 ± 4.33E-05	1.13E-02 ± 1.71E-04	2.44E-03 ± 6.10E-04	2.10E-03 ± 5.20E-04	2.78E-04 ± 1.54E-04	5.41E-03 ± 8.94E-04	7.92E-04 ± 1.34E-04	3.11E-04 ± 3.05E-05	2.17E-03 ± 7.71E-05
Rhizobiaceae	1.91E-02 ± 1.16E-03	2.98E-02 ± 1.19E-03	1.70E-02 ± 6.13E-04	1.99E-02 ± 1.38E-03	2.65E-02 ± 1.51E-03	3.70E-02 ± 1.17E-03	3.41E-02 ± 1.97E-03	4.14E-02 ± 1.02E-03	4.01E-03 ± 3.19E-04	5.44E-02 ± 1.60E-03
Rhizobiales_Incertae_Sedis	3.35E-04 ± 5.54E-05	1.65E-03 ± 2.03E-04	2.64E-04 ± 6.34E-05	1.66E-03 ± 2.93E-04	1.76E-04 ± 9.06E-05	1.70E-03 ± 1.36E-04	4.51E-04 ± 1.18E-04	8.17E-04 ± 2.24E-04	0.00E00 ± 0.00E00	1.34E-03 ± 2.11E-04
Rhodanobacteraceae	2.75E-04 ± 6.72E-05	3.12E-02 ± 1.50E-03	6.72E-04 ± 5.85E-05	9.30E-02 ± 7.30E-03	6.50E-04 ± 4.48E-05	9.16E-02 ± 6.16E-03	6.97E-04 ± 4.13E-04	6.94E-02 ± 4.09E-03	0.00E00 ± 0.00E00	6.11E-03 ± 4.74E-04
Solimonadaceae	0.00E00 ± 0.00E00	1.05E-03 ± 7.15E-05	1.19E-03 ± 1.69E-04	2.35E-02 ± 2.35E-03	1.19E-04 ± 8.63E-05	8.18E-03 ± 7.93E-04	8.71E-04 ± 2.09E-04	9.00E-03 ± 4.07E-04	1.60E-05 ± 1.60E-05	1.08E-03 ± 3.90E-05
Sphingobacteriaceae	2.32E-02 ± 3.17E-04	4.52E-03 ± 5.79E-04	7.24E-02 ± 3.75E-03	8.52E-03 ± 1.06E-03	2.57E-02 ± 2.34E-03	3.94E-03 ± 6.49E-04	1.55E-02 ± 1.47E-03	8.95E-03 ± 1.61E-03	1.44E-02 ± 1.31E-03	6.03E-02 ± 5.48E-04
Xanthobacteraceae	5.40E-04 ± 5.44E-05	2.25E-03 ± 2.72E-05	5.83E-03 ± 2.08E-04	1.33E-02 ± 3.91E-04	5.09E-04 ± 4.53E-05	4.00E-03 ± 4.23E-04	2.40E-03 ± 2.19E-04	3.17E-03 ± 3.09E-04	2.80E-04 ± 2.80E-04	1.37E-04 ± 7.48E-05
Xanthomonadaceae	1.02E-02 ± 9.95E-04	1.37E-02 ± 2.38E-04	1.13E-02 ± 3.39E-04	1.67E-02 ± 1.07E-03	2.51E-02 ± 1.02E-03	2.29E-02 ± 1.14E-03	1.40E-02 ± 8.09E-04	2.37E-02 ± 8.36E-04	2.32E-05 ± 2.32E-05	3.15E-02 ± 1.27E-03

Supplementary Table S5. Significantly altered fungal families (ITS2 gene) due to treatment (maturation, blending, sieving or acidification) of the composts. (A) Relative abundances (percentages) ± standard error of significantly altered fungal families between matured and immature composts in at least four compost batches. (B) Relative abundances (percentages) ± standard error of significantly altered fungal families between matured and acidified composts in at least four compost batches.

A.

	**Compost 1**		**Compost 2**		**Compost 3**		**Compost 4**		**Compost 5**	
	**Immature**	**Matured**	**Immature**	**Matured**	**Immature**	**Matured**	**Immature**	**Matured**	**Immature**	**Matured**
Helotiaceae	1.41E-05 ± 1.41E-05	6.96E-03 ± 5.68E-03	0.00E00 ± 0.00E00	7.57E-04 ± 7.18E-04	6.96E-06 ± 5.16E-06	3.41E-04 ± 1.81E-04	3.50E-06 ± 3.50E-06	4.67E-03 ± 1.57E-03	0.00E00 ± 0.00E00	1.55E-02 ± 8.12E-03
Hypocreaceae	0.00E00 ± 0.00E00	5.15E-03 ± 4.54E-03	0.00E00 ± 0.00E00	7.72E-04 ± 7.55E-04	7.53E-05 ± 3.91E-05	2.96E-03 ± 2.74E-03	0.00E00 ± 0.00E00	1.06E-03 ± 2.99E-04	0.00E00 ± 0.00E00	9.33E-03 ± 2.74E-03

B.

	**Compost 1**		**Compost 2**		**Compost 3**		**Compost 4**		**Compost 5**	
	**Matured**	**Acidified**	**Matured**	**Acidified**	**Matured**	**Acidified**	**Matured**	**Acidified**	**Matured**	**Acidified**
Eremomycetaceae	0.00E00 ± 0.00E00	2.33E-03 ± 8.87E-04	3.26E-03 ± 1.49E-03	2.48E-03 ± 1.21E-03	0.00E00 ± 0.00E00	9.22E-05 ± 6.11E-05	0.00E00 ± 0.00E00	4.47E-04 ± 3.38E-04	0.00E00 ± 0.00E00	4.50E-04 ± 2.48E-04
Onygenales_fam_Incertae_sedis	2.18E-03 ± 7.68E-04	1.20E-02 ± 3.58E-03	2.76E-04 ± 1.24E-04	5.27E-04 ± 2.33E-04	8.81E-04 ± 3.46E-04	1.63E-01 ± 5.05E-02	4.26E-05 ± 2.84E-05	6.99E-04 ± 3.80E-04	6.18E-05 ± 4.30E-05	3.19E-02 ± 2.19E-02

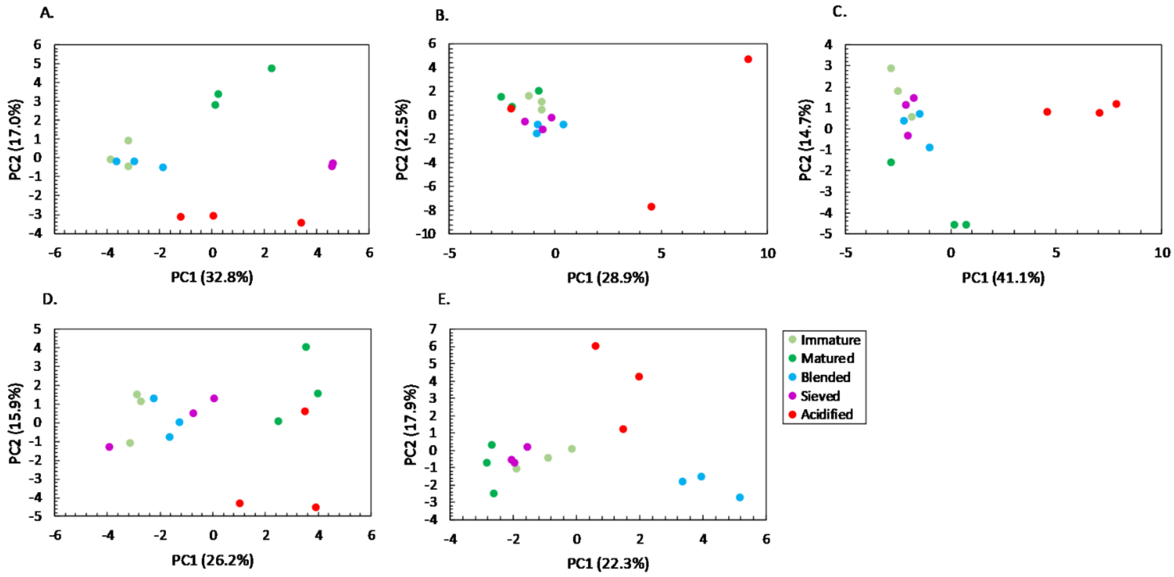

Supplementary Figure S6. Differentiation of immature, matured, blended, sieved and acidified composts based on carbon source metabolization profiles in (A) compost batch 1, (B) compost batch 2, (C) compost batch 3, (D) compost batch 4, (E) compost batch 5.

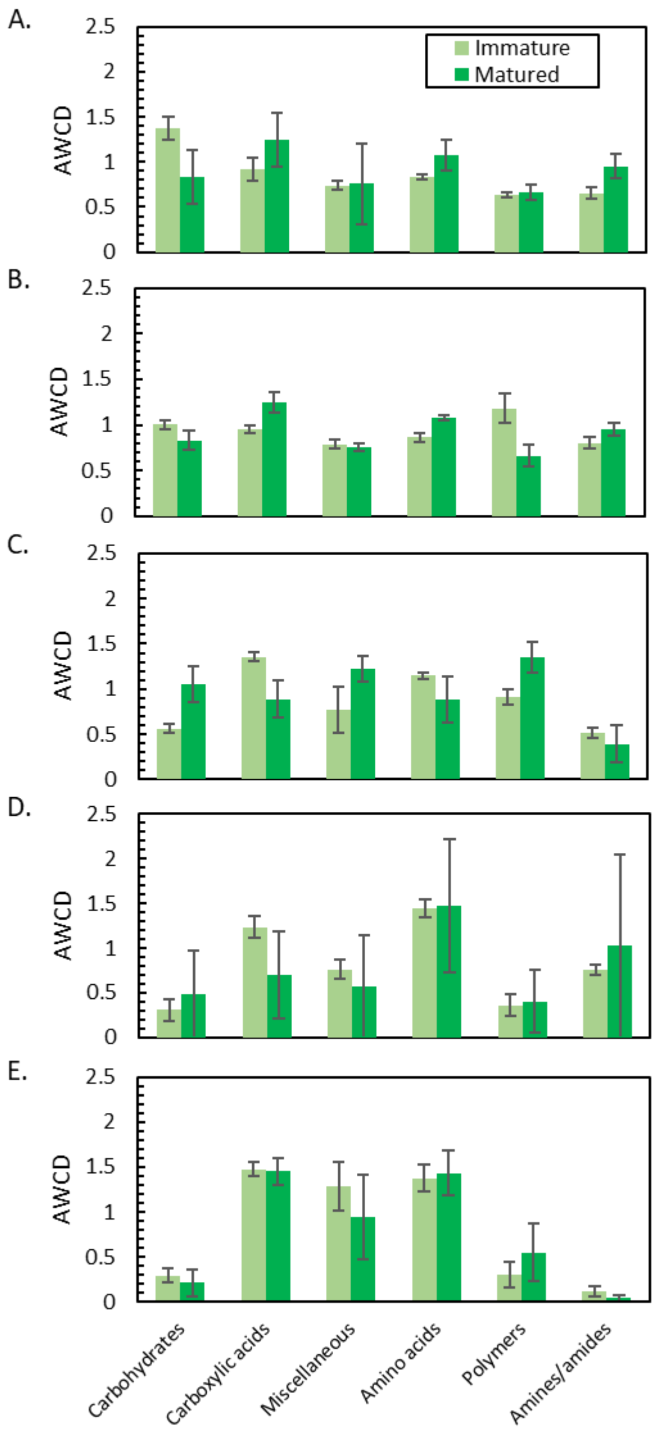

Supplementary Figure S7. Utilization (expressed as AWCD) of 31 carbon sources in Biolog Ecoplates grouped for class of substances by microbial communities of immature and matured composts in (A) compost batch 1, (B) compost batch 2, (C) compost batch 3, (D) compost batch 4, (E) compost batch 5.

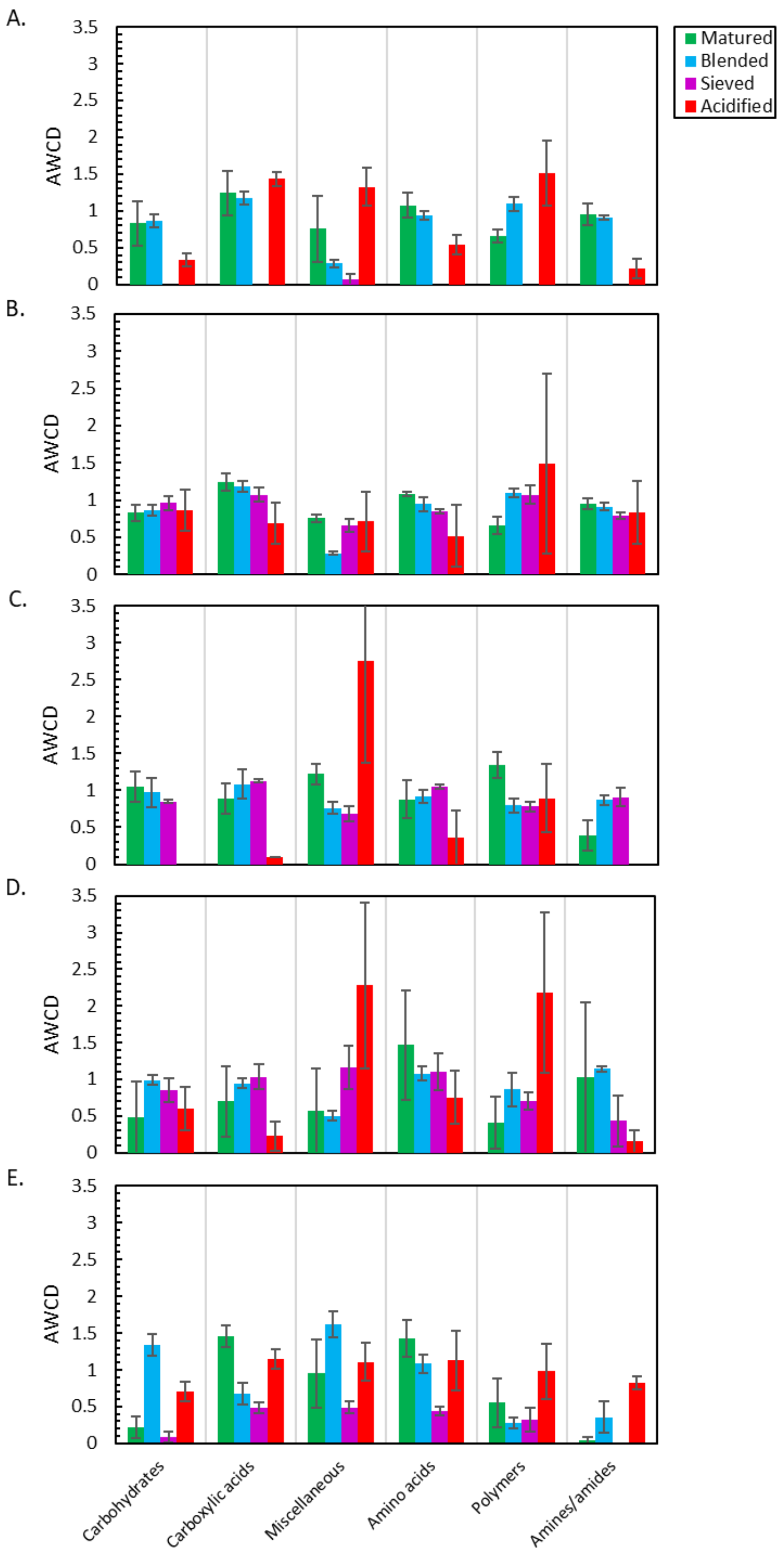

Supplementary Figure S8. Utilization (expressed as AWCD) of 31 carbon sources in Biolog Ecoplates grouped for class of substances by microbial communities of matured, blended, sieved and acidified composts in (A) compost batch 1, (B) compost batch 2, (C) compost batch 3, (D) compost batch 4, (E) compost batch 5.
